# Assessing Lettuce Exposure to a Multi-Pharmaceutical Mixture in Soil: Insights from LC-ESI-TQ Analysis and the Impact of Biochar on Pharmaceutical Bioavailability

**DOI:** 10.1021/acsomega.4c05831

**Published:** 2024-09-04

**Authors:** Jan Fučík, Vojtěch Jašek, Marie Hamplová, Jitka Navrkalová, Helena Zlámalová Gargošová, Ludmila Mravcová

**Affiliations:** † Institute of Chemistry and Technology of Environmental Protection, Faculty of Chemistry, Brno University of Technology, Purkyňova 118, 612 00 Brno, Czech Republic; ‡ Institute of Materials Chemistry, Faculty of Chemistry, Brno University of Technology, Purkyňova 118, 612 00 Brno, Czech Republic

## Abstract

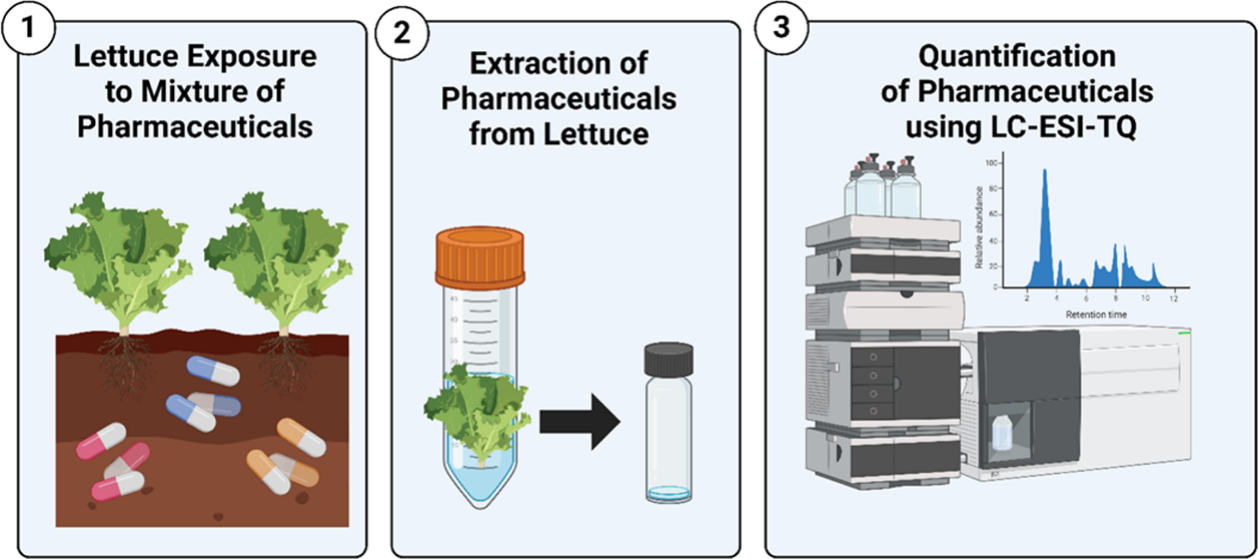

Agricultural practices introduce pharmaceutical (PhAC) residues into the terrestrial environment, potentially endangering agricultural crops and human health. This study aimed to evaluate various aspects related to the presence of pharmaceuticals in the lettuce-soil system, including bioconcentration factors (BCFs), translocation factors (TFs), ecotoxicological effects, the influence of biochar on the PhAC bioavailability, persistence in soil, and associated environmental and health risks. Lettuce (*Lactuca sativa* L.) was exposed to a mixture of 25 PhACs in two scenarios: initially contaminated soil (ranging from 0 to 10,000 ng·g^–1^) and soil irrigated with contaminated water (ranging from 0 to 1000 μg·L^–1^) over a 28-day period. The findings revealed a diverse range of BCFs (0.068–3.7) and TFs (0.032–0.58), indicating the uptake and translocation potential of pharmaceuticals by lettuce. Significant ecotoxicological effects on *L. sativa*, including weight change and increased mortality, were observed (*p* < 0.05). Interestingly, biochar did not significantly affect PhAC uptake by *L. sativa* (*p* > 0.05), while it significantly influenced the soil degradation kinetics of 12 PhACs (*p* < 0.05). Additionally, the estimated daily intake of PhACs through the consumption of *L. sativa* suggested negligible health risks, although concerns arose regarding the potential health risks if other vegetable sources were similarly contaminated with trace residues. Furthermore, this study evaluated the environmental risk associated with the emergence of antimicrobial resistance (AMR) in soil, as medium to high. In conclusion, these findings highlight the multifaceted challenges posed by pharmaceutical contamination in agricultural environments and emphasize the importance of proactive measures to mitigate the associated risks to both environmental and human health.

## 1 Introduction

Pharmaceuticals (PhACs), including veterinary antibiotics, have become widespread in agricultural fields through various contamination pathways. These include irrigation with treated wastewater, where PhAC concentrations range from hundredths of ng·L^–1^ to hundreds of μg·L^–1^^1,2^ as well as the application of biosolids and animal manures, where PhAC concentrations range from hundredths of ng·g^–1^ to thousands of μg·g^–1^.^3−7^ The escalating water scarcity, driven by urbanization and climate change, particularly affects arid and semiarid regions, leading to an increased reliance on treated wastewater for agricultural irrigation.^8,9^ Currently, the incorporation of biosolids and animal manure onto agricultural land is increasingly promoted within the circular economy framework as a viable alternative to mineral fertilizers.^10^ Globally, agricultural irrigation utilizes approximately 5.6 billion cubic meters (m^3^) of wastewater, with a continuous upward trend driven by worsening water scarcity and persistent drought conditions. Similarly, biosolids generation remains substantial, with 7.2 million tonnes produced annually in the United States and 4.7 million tonnes in Europe, figures expected to rise with population growth and urbanization.^11^ Despite the undeniable benefits of adopting a circular economy approach, indirect consequences must be considered, particularly the potential dissemination of both organic and inorganic micropollutants and pathogenic organisms into the soil environment.^10,12^ This can lead to contamination of soil by PhACs in concentrations ranging from hundredths of ng·g^–1^ to hundreds of μg·g^–1^.^5,13,14^ PhACs can profoundly influence the development and growth of plants, soil organisms, and microorganisms. Furthermore, they can be absorbed by soil organisms or agricultural crops, thereby entering the food chain and potentially impacting human populations. Another significant concern is the development of antimicrobial resistance.^15^

Recent environmental studies have extensively examined the uptake of pharmaceuticals by various terrestrial plants, including lettuce (*Lactuca sativa* L.),^11,16−18^ cowpea (*Vigna unguiculata* (L.) *Walp.*), turnip (*Brassica rapa* var. *Rapa L.*), basil (*Ocimum basilicumL.*),^16^ radish (*Raphanus sativusL.*),^19^ parsley (*Petroselinum crispum* (Mill.) *Fuss*),^20^ spinach (*Spinacia oleraceaL.*),^12^ carrot (*Daucus carotaL.*) and sweet potatoes (*Ipomoea batatas* (L.) *Lam.*).^21^ These studies typically involve experiments with pharmaceutical mixtures containing a few compounds, with the most common being up to 15^22^ and rarely up to 25 compounds.^12^ Investigations are commonly conducted on experimental fields following the application of biosolids, animal manure, or wastewater irrigation,^23,24^ or under laboratory conditions where crops are grown in initially contaminated soil, irrigated with contaminated water, or in amended soil, usually at only a single or just a few concentrations.^5,17,22,25,26^ Moreover, some studies^27,28^ are also conducted with plants under hydroponic conditions. Nevertheless, bioconcentration and translocation factors are typically determined from single concentrations as the ratio of roots/soil and leaves/roots, respectively,^12,29^ rather than from a range of concentrations as the slope of linear regression. Additionally, many studies have only analyzed plant leaves/shoots, neglecting both roots and soil samples and/or analyzing samples at the end of the experiment,^11,16,17^ providing only limited information on the potential uptake, accumulation, and translocation in plants. Some studies have focused solely on the quantification of pharmaceuticals in plants,^11,16^ while others have only concentrated on the ecotoxicological aspects of pharmaceuticals in soil environments,^30−33^ and occasionally both quantification and phytotoxicity have been evaluated within a single study.^17,29^ Furthermore, some studies have efficiently used biochar to reduce the bioavailability of pharmaceuticals by plants,^19,34,35^ although negative aspects such as prolonged half-lives of pharmaceuticals in soil should be assessed as well. Additionally, some studies have calculated the estimated daily intake of pharmaceutical residues through vegetables,^36,37^ although these data were not supplemented with an environmental assessment of the risk toward the emergence of antimicrobial resistance, which would essentialy provide a complete overview of the issue.

The objective of this study was to comprehensively assess the bioconcentration factors (BCFs) and translocation factors (TFs) of *L. sativa*, a model plant, under environmentally relevant conditions. Lettuce plants were cultivated in soil initially contaminated with concentrations ranging from 0 to 10,000 ng·g^–1^ and irrigated with water contaminated with concentrations ranging from 0 to 1000 μg·L^–1^, covering both environmentally relevant ranges and concentrations one magnitude higher.

Unlike previous studies,^12,29^ BCFs and TFs were determined from these concentration ranges, employing a novel approach of time-weighted average soil concentration for 25 pharmaceuticals, thus providing a better reflection of real-world conditions and examining a higher number of pharmaceuticals than typically considered in other research. Additionally, experiments were conducted in both nonamended and biochar-amended soil, with analyses of lettuce roots, leaves, and soil samples carried out on days 14, 21, 28, and 35, providing comprehensive information on the potential uptake, accumulation, translocation in plants, and persistence of pharmaceuticals in soil. Moreover, the phytotoxicity of *L. sativa* due to the presence of pharmaceutical contaminants was evaluated, considering parameters such as mortality rate and aboveground biomass weight change, with statistical tools applied to assess the significance of the obtained results. Finally, the estimated daily intake for humans was evaluated due to contamination of *L. sativa* through various routes (initially contaminated soil and irrigation with contaminated water) at two environmentally relevant concentrations, followed by a risk assessment regarding the emergence of antimicrobial resistance in the soil environment. Hence, unlike previous studies, this research provides comprehensive insights into the uptake of a wide range of pharmaceuticals by *L. sativa*, incorporating various pharmaceutical concentrations, contamination routes, biochar amendments, ecotoxicological results, and risk assessments within a single study.

## 2 Materials and Methods

### 2.1 Chemicals and Standards

Ethylenediamine tetraacetic acid (EDTA, ≥99%), citric acid monohydrate (≥99%), disodium hydrogen phosphate dodecahydrate (≥99%), and sodium sulfate anhydrous (≥95%), potassium dihydrogen phosphate (≥99%) and hydrochloric acid (35%) were purchased from Lach:ner (Czech Republic). Magnesium nitrate hexahydrate (>99%), ammonium (25%), methanol (LC-MS grade), acetonitrile (LC-MS grade), and water (LC-MS grade) were purchased from VWR. Sodium hydroxide (>98%) and phosphoric acid (85%) were purchased from Penta Chemicals (Czech Republic). Formic acid (LC-MS grade) and sodium chloride (>99%) were purchased from Sigma Aldrich (Germany).

The following pharmaceuticals (see their properties in Table S1) were used: acebutolol hydrochloride (≥99%), doxycycline hyclate (≥95%), oxytetracycline hydrochloride (≥94%) and sulfacetamide (≥98%) were purchased from Honeywell. Ofloxacin (≥98%) and sulfamethoxypyridazine (≥97%) were purchased from Thermo Fisher Scientific. Azithromycin (≥98%), chlortetracycline hydrochloride (≥91%), ciprofloxacin (≥98%), clarithromycin (≥97%), enrofloxacin (≥99%), erythromycin (≥97%), moxifloxacin (≥96%), norfloxacin (≥98%), pefloxacin mesylate dihydrate (≥97%), roxithromycin (≥95%), sulfadiazine (≥99%), sulfadimethoxine (≥98%), sulfamerazine (≥99%), sulfamethazine (≥99%), sulfamethoxazole (≥98%), sulfapyridine (≥99%), sulfathiazole (≥99%), tetracycline (≥98%) and trimethoprim (≥98%) were purchased from Sigma Aldrich (Germany). The following substances were used as internal standards (IS): ciprofloxacin-*d*^8^ (≥99%), enrofloxacin-*d*^5^ (≥99%), spiramycin (≥90%) and trimethoprim-*d*^9^ (≥97%) were purchased from Sigma Aldrich (Germany). Sulfamethoxazole-*d*^4^ was purchased from Neochema GmbH (Germany). Sulfathiazole-*d*^4^ was purchased from Toronto Research Chemicals (Canada).

Nitrogen gas (4.7) and Argon gas (5.0) were purchased from SIAD Czech spol. s.r.o. (Czech Republic). Nylon syringe filters (13 mm, 0.22 μm) and solid phase extraction (SPE) HLB cartridges (200 mg/6 mL, particle diameter 25–35 μm) were purchased from Chromservis (Czech Republic). For QuEChERS, dispersive SPE (dSPE): DSC-18 SPE, and PSA SPE were purchased from Sigma Aldrich (Germany).

### 2.2 Uptake of Pharmaceuticals by Lettuce, Concentration Range

Two sets of exposure experiments were carried out. In case A, the soil was initially contaminated with PhACs and irrigated with noncontaminated tap water. In case B, the soil was initially uncontaminated and irrigated with tap water contaminated with PhACs. Specifically, the soil was spiked with a mixture of 25 pharmaceuticals at concentrations ranging from 0 to 10,000 ng·g^–1^ dw of soil (physicochemical properties of soil provided in Table S2). In the other scenario, lettuce was daily soil-surface-irrigated with 25 mL of contaminated tap water at concentrations ranging from 0 to 1000 μg·L^–1^.

This study was carried out in a grow box (Green-Qube 1020L) under controlled conditions with a 16 h photoperiod (17,500 lx; LED panel: ViparSpectra XS2000 230W), air temperature of 23 ± 1 °C, and air humidity of 45 ± 5%. A single extraction fan and two oscillating fans were placed in the grow box to ensure appropriate air exchange and flow. To each dark PET pot (diameter of 95 mm; height of 80 mm; without drainage), 500 ± 1 g dw of soil was weighed. Similarly to studies,^38,39^*L. sativa* seeds were germinated in Petri-dishes on cotton wool, moistened with distilled water (without exposure to pharmaceuticals, at temperature of 23 °C). After 5 days, four sprouted seedlings were planted in each soil pot, and five replicates of each experiment were prepared. Initially, dry soil was watered with tap water to 40% MWHC (maximum water holding capacity) and watered daily to keep the soil humidity constant during the uptake experiments. The water-soluble organic liquid fertilizer (Natura, Czech Republic) with N-P-K 6.4–1.7–9.0 was used weekly as recommended. The pots were randomly placed into the grow box, and the pot positions were changed every third day to compensate for differences in light intensity. The lettuces were harvested after 14 and 28 days of exposure for both control and contaminated group. Lettuce samples were obtained by harvesting three lettuces from each pot after 14 days to evaluate ecotoxicological end points such as mortality rate and above-ground biomass weight. A single lettuce plant was sampled after 28 days to assess above-ground biomass weight and PhAC concentration both in roots and leaves. The obtained samples of leaves and roots were washed in deionized water to remove PhAC residues from the surface. Soil samples were obtained at sampling times of 0, 14, 21, 28, and 35 days.

Data measured in this experiment, specifically soil concentrations, were used to calculate degradation rate kinetics (eq 2). Average soil concentrations were then used to calculate degradation rates for both initially contaminated soil (eq 3) and soil irrigated with contaminated water (eq 4). Consequently, BCFs were calculated using measured concentrations in lettuce roots along with the determined average soil concentrations (eq 1). Finally, TFs were calculated as the ratios of pharmaceutical concentrations in lettuce leaves to those in lettuce roots (eq 5). The results of this experiment are shown in Table 1 and discussed in detail in Subsection 3.1 of the manuscript. Moreover, ecotoxicological data are evaluated in Subsection 3.3 and illustrated in Figures 3–6.

**Table 1:** Results of Pharmaceutical Uptake by *L. sativa*—Bioconcentration Factors (BCFs), Translocation Factor (TF), Degradation Rate Kinetics (*k*) and Respectively Their Standard Errors (SE) for Individual Pharmaceuticals after 28 Days of PhAC Uptake in Non-amended and Soil Biochar-amended (N.D. – Not Determined)

		biocconcentration and translocation factor in Biochar nonamended soil										nonamended soil		biochar-amended soil	
pharmaceutical group	pharmaceutical name	BCF_soil_ [−]	SE (BCF_soil_) [−]	TF_soil_ [−]	SE (TF_soil_) [−]	linearity range [ng·g^–1^]	BCF_irrigation_ [−]	SE (BCF_irrigation_) [−]	TF_irrigation_ [−]	SE (TF_irrigation_) [−]	linearity range [μg·L^–1^]	*k* [d^-1^]	SE (k) [d^–1^]	*k* [d^–1^]	SE (*k*) [d^–1^]
β-blockers	acetobutolol	0.319	0.024	0.132	0.012	5000	2.32	0.17	0.15	0.04	1000	0.036	0.017	0.071	0.005
fluoroquinolones	ciprofloxacin	0.85	0.05	0.088	0.015	5000	1.90	0.07	0.130	0.019	1000	0.099	0.005	0.075	0.012
	enrofloxacin	0.76	0.09	0.032	0.003	5000	2.37	0.10	N.D.		1000	0.0092	0.0003	0.0157	0.0009
	moxifloxacin	0.347	0.021	0.16	0.04	5000	2.2	0.3	0.26	0.04	1000	0.013	0.003	0.012	0.003
	norfloxacin	0.082	0.011	N.D.		3000	1.08	0.09	N.D.		1000	0.0115	0.0014	0.0133	0.0020
	ofloxacin	0.264	0.003	0.09	0.04	3000	1.56	0.15	0.178	0.015	1000	0.0168	0.0018	0.0094	0.0019
	pefloxacin	0.84	0.03	0.07	0.03	10,000	3.7	0.3	0.31	0.05	1000	0.116	0.017	0.0128	0.0015
macrolides	azithromycin	0.184	0.013	0.057	0.016	5000	1.02	0.05	0.17	0.04	1000	0.00076	0.00020	0.0129	0.0012
	clarithromycin	0.30	0.03	0.033	0.008	5000	0.69	0.10	0.267	0.003	1000	0.044	0.011	0.013	0.006
	erythromycin	0.88	0.06	0.56	0.15	3000	3.2	0.7	0.065	0.004	1000	0.073	0.003	0.019	0.006
	roxithromycin	0.372	0.024	0.34	0.16	5000	0.926	0.011	N.D.		1000	0.098	0.011	0.023	0.008
sulfonamides	sulfacetamide	0.31	0.04	N.D.		5000	1.5	0.3	N.D.		1000	0.198	0.017	0.113	0.017
	sulfadiazine	0.17	0.04	N.D.		5000	0.88	0.12	N.D.		1000	0.0994	0.0015	0.082	0.005
	sulfadimethoxine	0.89	0.11	N.D.		10,000	1.45	0.24	N.D.		1000	0.050	0.011	0.042	0.014
	sulfamerazine	0.53	0.07	N.D.		10,000	N.D.		N.D.		1000	0.055	0.009	0.088	0.021
	sulfamethazine	0.39	0.04	0.16	0.06	10,000	N.D:		N.D.		1000	0.052	0.005	0.125	0.012
	sulfamethoxazole	0.78	0.19	N.D.		3000	1.69	0.21	0.13	0.02	1000	0.039	0.003	0.08	0.03
	sulfamethoxypyridazine	0.245	0.017	0.28	0.06	5000	0.41	0.06	N.D.		1000	0.074	0.004	0.055	0.003
	sulfapyridine	0.278	0.018	0.118	0.019	10,000	0.87	0.21	N.D.		1000	0.13	0.03	0.117	0.019
	sulfathiazole	0.13	0.05	N.D.		5000	0.14	0.03	N.D.		1000	0.133	0.012	0.090	0.004
	trimethoprim	0.17	0.03	0.28	0.04	3000	1.19	0.11	0.13	0.02	1000	0.022	0.003	0.094	0.008
tetracyclines	chlortetracycline	0.068	0.004	0.42	0.06	5000	0.708	0.011	0.35	0.06	1000	0.037	0.003	0.023	0.004
	doxycycline	0.34	0.03	N.D.		5000	1.29	0.12	0.58	0.12	1000	0.030	0.003	0.0212	0.0022
	oxytetracycline	0.104	0.010	0.32	0.08	5000	1.21	0.06	N.D.		1000	0.028	0.004	0.0193	0.0022
	tetracycline	0.218	0.011	N.D.		10,000	1.53	0.22	N.D.		1000	0.03113	0.00017	0.020	0.003

### 2.3 Uptake of Pharmaceuticals by Lettuce, Effect of Biochar

The uptake experiment followed the methodology outlined in Subsection 2.2. The soil was deliberately contaminated with a pharmaceutical mixture at a concentration of 3000 ng·g^**–**1^ and irrigated with uncontaminated tap water. The experiment involved both nonamended soil and soil amended with biochar at a concentration of 2% by weight. Detailed physicochemical properties of the biochar are provided in Table S3 from the study.^40^ Lettuce samples were collected by harvesting three seedlings from each pot after 14 days to evaluate ecotoxicological end points and PhAC concentrations in the entire seedlings (roots and leaves were not separated due to the small size of *L. sativa*). Subsequently, individual lettuces were sampled after 21, 28, and 35 days to assess the above-ground biomass weight and PhAC concentration in both roots and leaves separately. The collected seedlings, leaves, and roots were washed with deionized water to remove antibiotics from the surface, then extracted and analyzed. Additionally, soil samples were obtained at sampling times of 0, 14, 21, 28, and 35 days.

**Figure 1:**
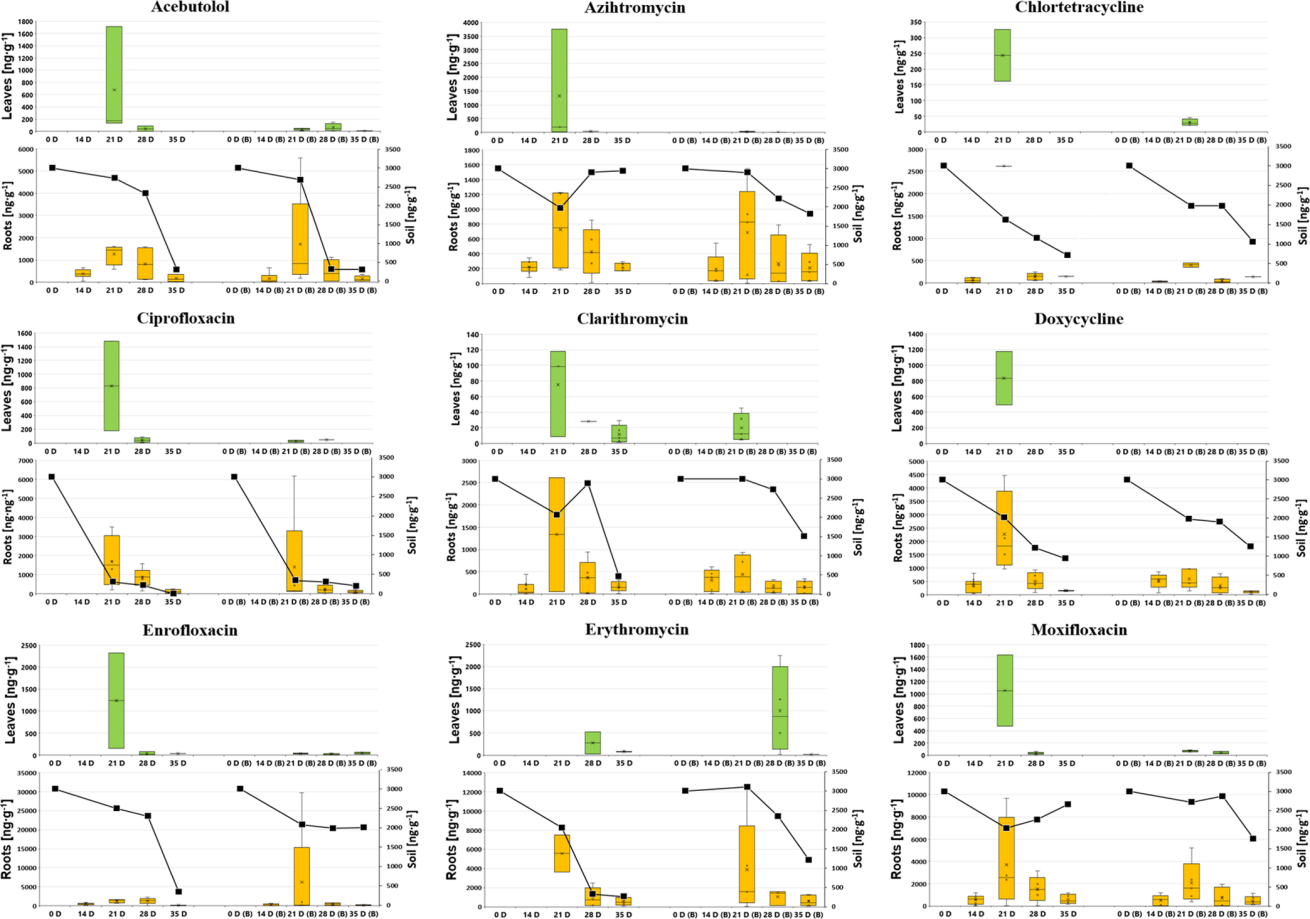
Dynamics of individual PhAC concentrations in lettuce leaves (upper, green box plot) and roots (lower, orange box plot) in [ng·g^–1^ dw], alongside soil concentrations (lower graph, black line chart on right *Y*-axis in ng·g^–1^ dw) over 35 days: comparison between nonamended soil (on the left side of each figure) and biochar-amended soil (designated as “B” on the right side of each figure).

**Figure 2:**
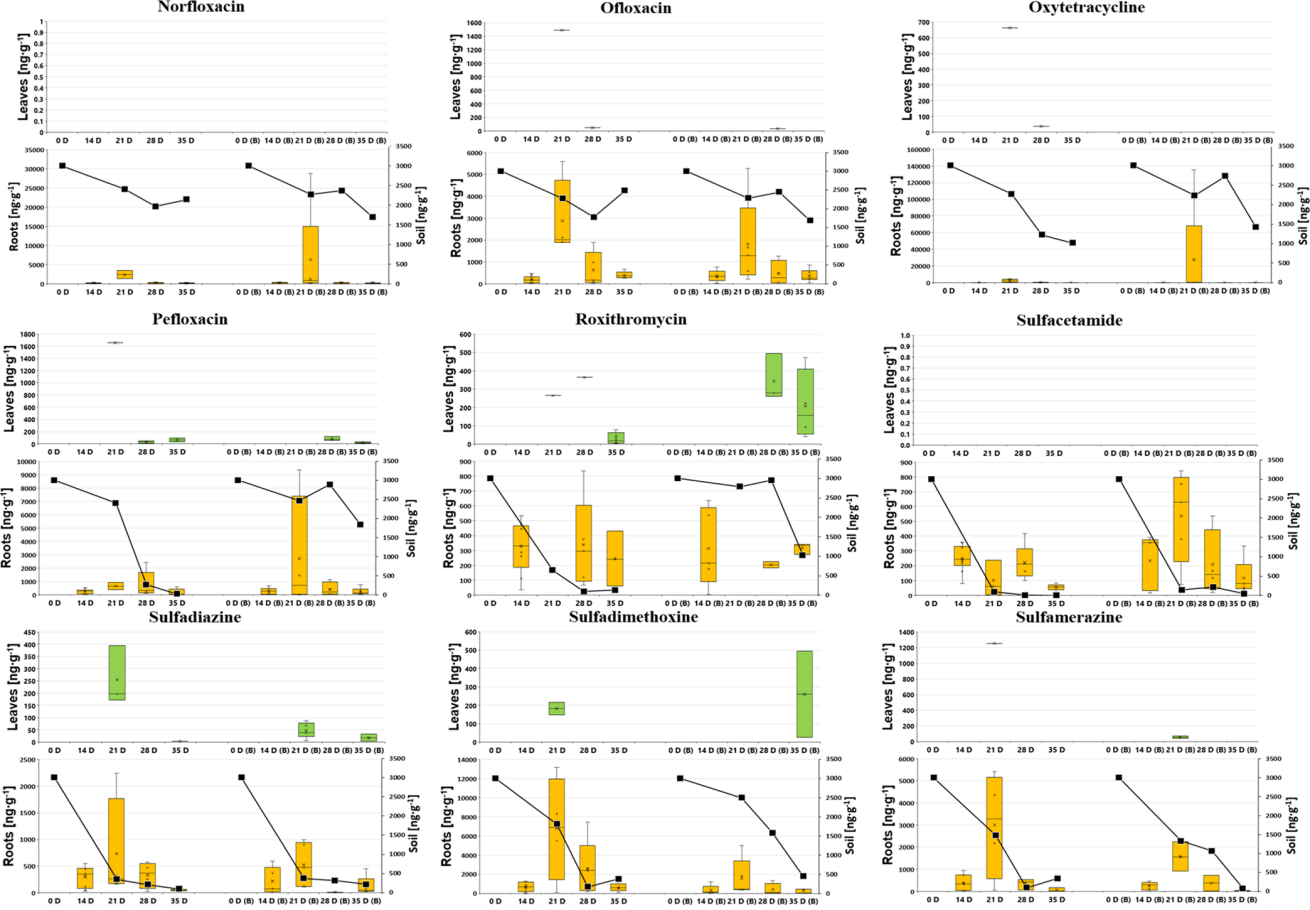
Dynamics of individual PhAC concentrations in lettuce leaves (upper, green box plot) and roots (lower, orange box plot) in [ng·g^–1^ dw], alongside soil concentrations (lower graph, black line chart on right *Y*-axis in ng·g^–1^ dw) over 35 days: comparison between nonamended soil (on the left side of each figure) and biochar-amended soil (designated as “B” on the right side of each figure).

**Figure 3:**
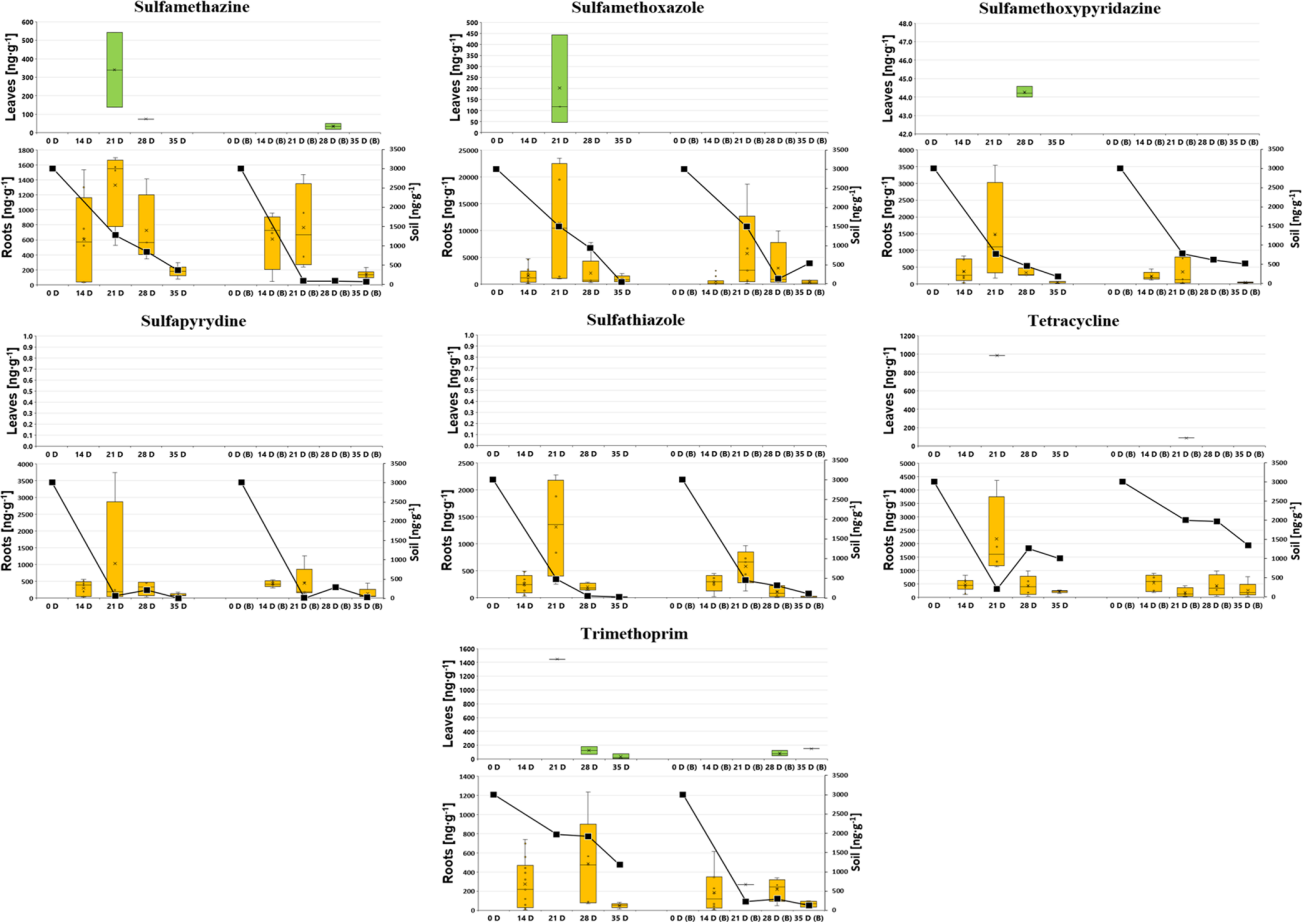
Dynamics of individual PhAC concentrations in lettuce leaves (upper, green box plot) and roots (lower, orange box plot) in [ng·g^–1^ dw], alongside soil concentrations (lower graph, black line chart on right *Y*-axis in ng·g^–1^ dw) over 35 days: comparison between nonamended soil (on the left side of each figure) and biochar-amended soil (designated as “B” on the right side of each figure).

Data measured in this experiment, specifically soil concentrations, were used to calculate degradation rate kinetics in both nonamended soil and biochar-amended soil (eq 2), as shown in Table 1. Consequently, concentrations in lettuce leaves, lettuce roots, and soil were plotted in Figures 1, 2, 3 and S1, and were discussed in detail in Subsection 3.4 of the manuscript. Moreover, ecotoxicological data are evaluated in Subsection 3.3 and illustrated in Figure 7.

### 2.4 Extraction of Pharmaceuticals from Lettuce and Soil Samples

We used extraction methods developed and validated by study^41^ for the quantification of PhACs in lettuce leaves, lettuce roots, and soil samples. Specifically, the lettuce samples were extracted using the QuEChERS-based method, while the soil samples were processed with our ultrasound-assisted extraction approach, followed by solid-phase extraction as described in study.^41^ Detailed protocols for these methods can also be found in the Supporting Information, Appendix 1. The resulting extracts from all sample types were analyzed using the liquid chromatography-mass spectrometry/MS (LC-MS/MS) method, which is outlined in the same publication^41^ and further detailed in the Supporting Information, Appendix 2.

### 2.5 Statistical Analysis

Statistical analyses were conducted using Python and GraphPad Prism (version 10.2.0). A *p*-value of 0.05 was considered indicative of statistical significance for all tests. Specifically, *t* tests were employed to assess whether BCFs and TFs for each drug varied based on the contamination route (lettuces grown in initially contaminated soil vs lettuces irrigated with contaminated water). Additionally, *t* tests were used to evaluate whether biochar influenced the bioavailability of pharmaceuticals for uptake by *L. sativa* and affected the persistence of pharmaceuticals in the soil environment. ANOVA followed by Dunnett’s test was used to analyze ecotoxicological results, such as biomass weight, and box plots were created using GraphPad Prism software. Furthermore, heat maps depicting *L. sativa* pharmaceutical concentrations were visualized using the MetaboAnalyst 6.0 web-based statistical platform.^42^

## 3 Results and Discussion

### 3.1 Lettuce Exposure to Different Concentrations of Pharmaceuticals

The *L. sativa* seedlings were exposed to a mixture of 25-PhACs representing various therapeutic classes. Two series of exposure experiments were carried out in a controlled laboratory setting, each lasting 28 days. In the first scenario (Case A), the soil was initially contaminated with PhACs and then irrigated with clean tap water. In the second scenario (Case B), the soil was not initially contaminated but was irrigated with tap water containing PhACs. To thoroughly evaluate bioconcentration factors (BCFs), the experiments involved a wide range of soil contamination with concentrations ranging from 0 to 10,000 ng·g^–1^ or irrigation with water contaminated at PhAC levels ranging from 0 to 1000 μg·L^–1^.

#### 3.1.1 Bioconcentration Factors—Calculation

BCFs within the plant–soil system were computed using eq 1, typically based on either the initial exposure soil concentrations^12,29,43,44^ or the time of sampling.^45^ However, relying on either can lead to underestimation or overestimation of BCFs, respectively, as neither accurately mirrors real-world conditions where PhAC degradation occurs. Degradation rates *k* [d^–1^] and their standard error (SE) were determined through linear regression using eq 2 for each individual substance in the lettuce-soil system (Table 1). PhAC degradation in soil followed first-order kinetics, in line with findings from prior research.^22^

To derive more accurate BCFs, time-weighted average (TWA) soil concentrations were calculated, for both initially contaminated soil (eq 3) and soil irrigated with pharmaceutical-contaminated water (eq 4). These TWA values were then used in eq 1 to compute the BCF values (Table 1), which were determined as the slope of the linear equation *y = ax* (*y* represents roots concentration, *x* represents TWA soil concentrations and *a* (the slope) corresponds to the BCF). While this methodology using TWA soil concentration has been suggested in prior studies,^46,47^ it has not yet been adopted for BCF determination in lettuce-soil system. By incorporating the time variable into the calculation, this approach provides a more nuanced and representative measure, particularly in situations where concentrations change over time. By accounting for changes in concentration throughout the exposure period, this method places greater emphasis on times when the compound levels are higher and over longer durations, leading to a more accurate average.
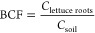
1where *c*_lettuce roots_ [ng·g^**–**1^] stands for the concentration in lettuce roots at the end of the experiment, while *c*_soil_ [ng·g^**–**1^] indicates the soil concentration, which could be the initial concentration, the concentration at the experiment’s conclusion, or the time-weighted average concentration.
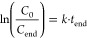
2where *c*_0_ [ng·g^**–**1^] represents the initial soil concentration, *c*_end_ [ng·g^**–**1^] is the concentration at the end of the experiment, *k* [d^–1^] indicates the degradation rate kinetics, and *t*_end_ [day] stands for the experiment’s duration.
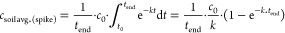
3where *c*_soil,avg. (spike)_ [ng·g^**–**1^] denotes the time-weighted average soil concentration throughout the experiment with initial soil contamination, *t*_end_ [day] stands for the experiment’s duration, *c*_0_ [ng·g^**–**1^] represents the initial soil concentration and *k* stands for the degradation rate kinetics.
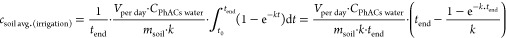
4where *c*_soil,avg. (irrigation)_ [ng·g^**–**1^] denotes the time-weighted average soil concentration of a contaminant in the soil throughout an experiment involving irrigation with contaminated water. The duration of the experiment is given by *t*_end_ [day], and the volume used daily for irrigation water used is represented by *V*_per day_ [L]. The concentration of pharmaceuticals in the irrigation water is denoted by *C*_PhACs water_ [μg·L^**–**1^]. The total dry weight of the soil in the PET container is given by *m*_soil_ [g], and *k* [d^–1^] indicates the rate constant for degradation or decay of the contaminant.

#### 3.1.2 Bioconcentration Factors

Following the experiments described in Subsection 2.2, the BCF > 1 indicates significant bioconcentration in lettuce roots, whereas a BCF < 1 suggest that the uptake occurs, although not at a significant rate. In agreement with studies,^48,49^ the bioconcentration of PhACs is well modeled using linear equations, with coefficients of determination *R*^2^ > 0.92 for all compounds in both contamination routes. This linearity indicates that the concentration in lettuce roots is proportional to the soil concentration (and alternatively to concentration in irrigation water, animal manure or biosolids), consistent with findings from other studies.^17,26^ However, the degree of linearity varied among the PhACs tested, as shown in Table 1, possibly because of several factors. These include higher metabolism rates when concentrations exceed certain thresholds, limitations in the linear model, inaccuracies in measuring high concentrations, uneven distribution of PhACs in soil, and inherent biological variability in *L. sativa*.

After 28 days of PhAC exposure and *L. sativa* growth, the determined BCF values (Table 1) for initially spiked soil and soil irrigated with PhAC-contaminated water were as follows: for β-blockers: 0.319 in spiked soil and 2.32 in soil irrigated with PhAC-contaminated water; for fluoroquinolones: 0.082 to 0.85 in spiked soil and 1.08 to 3.7 in irrigated soil; for macrolides: 0.184 to 0.88 in spiked soil and 0.69 to 3.2 in irrigated soil; for sulfonamides: 0.13 to 0.89 in spiked soil and 0.14 to 1.69 in irrigated soil; and for tetracyclines: 0.068 to 1.19 in spiked soil and 0.708 to 1.53 in irrigated soil. Furthermore, *t* test was performed to evaluate whether the BCF for each drug varied depending on the contamination route. It was found that BCF values were significantly higher (*p* < 0.05) for irrigation with contaminated water compared to initially spiked soil for 20 of 25 PhACs. The exceptions were sulfamerazine, sulfamethazine, sulfadimethoxine, sulfamethoxypyridazine, sulfapyridine, and sulfathiazole, all of which are soil-mobile sulfonamides.^50^ In contrast to our findings, a review^36^ reported that antibiotic concentrations in plants due to manure application and wastewater irrigation showed no significant differences (*p* > 0.05). This discrepancy could be explained by differences in the experimental design and data evaluation. Our study used TWA concentrations under controlled laboratory conditions to calculate BCFs, which may have led to different outcomes compared to studies involving field conditions or other methods of exposure. Nonetheless, the higher BCFs observed in our study could be attributed to continuous exposure and potential differences in soil microbiota activity due to the increased concentrations of pharmaceuticals in the initially spiked soil. Additionally, we calculated the TWA soil concentrations for both initially spiked soil and soil irrigated with contaminated water (Table S5), assuming a 28-day experiment duration with equal total amounts of pharmaceuticals introduced in each scenario. For all 25 pharmaceuticals analyzed, the TWA soil concentrations were higher in the initially spiked soil. This raises additional concerns about higher bioaccumulation factors when irrigation with contaminated water is used, as it suggests that continuous exposure may lead to increased bioavailability and uptake despite lower soil concentrations.

Nevertheless, similar BCFs were observed in *L. sativa* in a study by study,^44^ with atenolol, sotalol, and propranolol exhibiting BCF values between 0.082 and 0.504, depending on the specific compound and soil type. Additionally, another study^29^ reported comparable BCFs for sulfamethoxypyridazine, tetracycline, ofloxacin, norfloxacin, and difloxacin, with values ranging from 0.011 to 0.025. An extensive study^12^ explored BCFs for 25 different compounds in spinach, with values spanning from 0.16 to 37.85, varying based on the specific pharmaceutical, soil type, and soil amendment.

Sorption–desorption and transformation of PhAcs in soil systems are dynamic processes that work together to regulate the amount of pharmaceuticals available in soil pore water for plant uptake.^5^ Numerous studies have shown that various factors within the plant–soil-pharmaceutical system can affect drug uptake and, consequently, bioconcentration factors. Studies^12,37,48,51^ have highlighted the key physicochemical properties of PhACs that influence plant uptake, including molecular size, molecular weight, octanol–water partition coefficient (*K*_ow_), acid dissociation constant (p*K*_a_), water solubility, and the number of hydrogen bonds. Additionaly, studies^37,48^ have shown that uptake rates can vary among different plant species (or even cultivars or genotypes^18,29^) due to differences in plant physiology, root morphology, and other biological characteristics such as lipid and carbohydrate content. Studies^5,12,44,52,53^ have demonstrated that certain soil properties can significantly affect the uptake of PhACs by plants, such as the presence of soil microbes, divalent cations (Ca^2+^, Mg^2+^, etc.) organic matter content, clay mineral fraction, ion exchange capacity, and the soil-water distribution coefficient (*K*_d_), which can vary by 1–2 orders of magnitude among different soil types (as also shown in Table S7). Furthermore, studies 9,35 have highlighted the influence of environmental conditions, such as climate, temperature, photoperiod and humidity, on PhAC uptake. These factors impact soil moisture and microbial activity, which in turn influence drug availability to plants. Additionally, studies^12,39^ have discussed the impact of soil amendments (such as sewage sludge, animal manure, and wastewater irrigation) on the uptake of PhACs, noting that these amendments can alter the soil’s organic content and microbial activity, thereby affecting PhAC bioavailability. Finally, study^25^ explored the effect of irrigation methods, comparing soil surface irrigation with overhead irrigation. The findings showed similar BCFs for lettuce under both irrigation methods, suggesting that the mode of irrigation might have less impact on PhAC uptake compared to other factors mentioned.^25^ Additionally, studies^19,34,35,54^ have indicated that adding biochar to soil could effectively reduce plant uptake of various contaminants and their ecotoxicity.

#### 3.1.3 Translocation Factors

Similarly to BCFs, following the experiments described in Subsection 2.2, translocation factors (TFs) were calculated as the slope of a linear equation *y* = *ax*, (*y* represents the concentration in the leaves, *x* represents the concentration in the roots, and *a* (the slope) corresponds to the TF), as outlined in eq 5 and previous studies.^48,49^
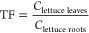
5where *c*_lettuce leaves_ [ng·g^–1^] stands for the concentration in lettuce leaves at the end of the experiment and *c*_lettuce roots_ [ng·g^–1^] stands for the concentration in lettuce roots at the end of the experiment.

PhACs with a TF < 1 are not readily transported from roots to leaves in plants, indicating that the most of the compound remains in the roots. In contrast, a TF > 1 implies efficient translocation from roots to leaves, indicating a higher potential for bioconcentration in aerial parts of the plant.^25^ The determined TFs (Table 1) for initially spiked soil and soil irrigated with PhAC-contaminated water were as follows: for β-blockers: 0.132 in spiked soil and 0.15 in soil irrigated with PhAC-contaminated water; for fluoroquinolones: 0.032 to 0.16 in spiked soil and 0.13 to 0.31 in irrigated soil; for macrolides: 0.033 to 0.56 in spiked soil and 0.065 to 0.267 in irrigated soil; for sulfonamides: 0.118 to 0.28 in spiked soil and 0.13 in irrigated soil; and for tetracyclines: 0.35 to 0.42 in spiked soil and 0.35 to 0.58 in irrigated soil. Additionally, a *t* test was conducted to determine wheter the TF of each drug varied depending on the contamination route (considering both treatments). The results revealed that TF values were significantly different (*p* < 0.05) for only 5 of 25 PhACs: azithromycin, clarithromycin, erythromycin, pefloxacin, and trimethoprim. In agreement with study,^25^ this indicates that translocation is not significantly influenced by the contamination route but is more likely driven by the concentration of pharmaceuticals in the plant’s roots.

In contrast to BCFs, TFs were not determined for all pharmaceutical or contamination route because many values were below the limit of detection. Furthermore, none of the TF values exceeded 1, indicating a low translocation from roots to leaves for the compounds tested. In the study,^25^ TF values were determined for 11 compounds in lettuce grown with surface soil irrigation. The TF values ranged from 0.07 to 8.15, depending on the specific drug and its therapeutic class. In another study,^49^ the TF for carbamazepine in pumpkin (*Cucurbita pepoL.*) reached 1.773. These studies illustrate that translocation can vary significantly across different compounds and plant species. In general, translocation in plants depends on various factors, such as plant type and physiology,^48^ physicochemical properties of PhACs,^37^ the surrounding environment and its impact on plant metabolism,^37^ and the concentration of pollutants in the environment/plant roots.^49^

### 3.2 Lettuce Exposure to Pharmaceuticals in Biochar-Amended and Non-Amended Soils

*L. sativa* plants were grown in the soil contaminated with a pharmaceutical mixture at a concentration of 3000 ng·g^–1^, with experimental conditions including both nonamended soil and soil amended with biochar at a 2% weight concentration. Apart from sampling on day 14, when the low weight of *L. sativa* seedlings required analysis of the whole plant, roots and leaves were analyzed separately. The experiment is described in detail in Subsection 2.3 of the manuscript.

The trends illustrated in Figures 1–3, along with the heatmap in Figure S1 for lettuce samples, indicate a consistent decrease in PhAC concentrations in both lettuce samples and surrounding soil over time. The declining concentrations in soil are a result of the degradation of pharmaceuticals according to first-order degradation kinetics. Within plant tissue, decreasing concentrations can be attributed to two factors: the dilution effect caused by plant growth and the reduction of soil concentrations over time, since plant uptake is proportional to the level of soil contamination. This trend of decreasing concentrations holds true regardless of whether the soil was amended with biochar or not, with the highest concentrations observed in lettuce roots on day 21 for the majority of PhACs. Consistent with Subsection 3.1 (as indicated by BCFs and TFs in Table 1) and previous studies,^20,36^*L. sativa* roots, like those of many other plants, typically show significantly higher detection frequency and PhAC concentration than leaves. This aligns with our findings, as illustrated in Figures 1–3 and S1. A review^36^ examined the concentration profile of different classes of antimicrobials and found that statistical analysis suggested significantly higher plant uptake of TCs compared to FQs and MLs (*p* < 0.05). In contrast, our results, which compared BBs, FQs, MLs, SAs, and TCs, did not reveal any statistically significant differences in uptake of *L. sativa* roots (*p* > 0.05).

Generally, comparing concentration trends over time with existing literature is challenging because few studies simultaneously analyze both root and shoot samples alongside soil samples over an extended period. Additionally, studies have been conducted under various conditions,which potentially influence pharmaceutical uptake and translocation. These differences, as discussed in Subsection 3.1, can complicate comparisons and may lead to differing results. Most studies usually conduct a single sampling at the end of the exposure experiment, and in many cases, only the shoots, as the edible part of plant or vegetable are analyzed.^11,16,17^ Nevertheless, a study^18^ analyzed concentrations of PhACs in lettuce leaves after 24, 35, and 46 days of exposure. The study found that PhAC concentrations in the final sampling (35 days) were lower than those in the previous sampling (21 days). This is similar to our findings, where the highest PhAC concentrations were observed on day 21. The observed decrease in concentrations over time can be attributed to the plant’s growth rate outpacing the rate of antimicrobial uptake, effectively diluting the concentration in the plant tissues. Furthermore, a study^55^ examined the uptake of veterinary antimicrobials (chlortetracycline, enrofloxacin, sulfathiazole) in radish at soil concentrations of 5 and 20 μg·g^–1^. Consistent with our results, this study found that antimicrobial concentrations in the soil decreased over time, with higher trace concentrations in roots than in leaves and more pronounced on day 30 than on day 45.

Additional study^11^ reported that the outer leaves of lettuce contained higher concentrations of carbamazepine and its metabolites compared to the inner sections. This difference is likely because the inner leaves are younger and more sheltered, resulting in less transpiration compared to the older outer leaves, which are more exposed to light, wind, and drier conditions.

#### 3.2.1 Phytoremediation Potential of Plants

While numerous studies^56−59^ have proposed that plants might be used successfully to remediate PhACs from soil, a study^17^ revealed that in pot experiments, plants generally absorbed less than 2% of the PhACs present in the soil. This suggests that although phytoremediation holds some potential, its effectiveness may be constrained by low plant uptake rates, unless hyperaccumulators are identified. In general, it is accepted that excluders, accumulators, and hyperaccumulators have BCF values of less than 1, greater than 1, and greater than 10, respectively, while accumulators and hyperaccumulators have TF values greater than 1.^60^ The mentioned thresholds and results in Table 1 suggest that *L. sativa* functions as an excluder when soil is initially contaminated with pharmaceuticla residues (BCFs < 1) and as an accumulator when the lettuces are irrigated with contamianted water (BCFs > 1 for the majority of pharmaceuticals). The patterns of soil concentrations shown in Figures 1–3 suggest that most parent compounds are degraded in the soil environment. However, these degradation products might also be absorbed by plants without being detected, or PhACs can be metabolized within plant tissue to form both biologically active and inactive compounds, thus avoiding detection as well. Generally, these degradation products and metabolites often remain unquantified, and in many cases, they are not even identified, complicating the evaluation of studies focused on the uptake and remediation of these compounds.^60,61^

Nevertheless, since soil microorganisms play a major role in removing pharmaceuticals, the composition of these microbial communities can be strongly influenced by the choice of plant species. This variability in microbial composition can impact the effectiveness of phytoremediation, even when direct uptake by the plant itself might not be a significant factor.^59,62^ Unlike studies focusing on organic micropollutants, phytoremediation investigations^63,64^ addressing heavy metal contamination entail a different approach. In these studies, the total concentration of heavy metals in the soil-plant system can be readily determined because there are no degradation products or metabolites to consider.

#### 3.2.2 Impact of Biochar on PhACs Uptake

Although many studies have reported that adding biochar to soil can increase the sorption of various heavy metals, pesticides, herbicides, and pharmaceuticals,^19,34,35^ our findings suggest that biochar’s effect (at 2% w/w) on the bioavailability of pharmaceuticals to *L. sativa* roots and shoots was negligible for all tested PhACs (*p* > 0.05). Consistent with our findings, a study^34^ reported that biochar amendment did not significantly affect PhAC concentrations in *L. sativa* roots. Meanwhile, a study^19^ that investigated radishes grown in biochar-amended, pharmaceutical-contaminated soil found that adding 1% biochar to the soil significantly reduced the uptake of 11 pharmaceuticals while increasing the uptake of lincomycin (a macrolide antibiotic). Similarly, a study^35^ found that adding 2.5% woody biochar to soil significantly reduced the uptake rate of sulfamethoxazole by water spinach (*Ipomoea aquaticaForssk.*) by 95%. The study also determined that sulfamethoxazole adsorption by soil and biochar-amended soil followed the Langmuir model, with maximum adsorption capacities of 0.718 and 3.448 mg·g^–1^, respectively, suggesting a substantial adsorption capacity relative to typical environmental concentrations.

Scientific studies^19,34,65^ have reported differing outcomes on the impact of biochar on the bioavailability of pharmaceuticals to plants. This variation can be attributed to several factors, including the rate of biochar amendment (ranging from 0.5 to 5.0%), the specific properties of the biochar and soil, soil microbiota, rhizobacteria, pharmaceutical characteristics, plant species (including plant microbiota), experimental conditions, or likely a combination of these elements. Incorporating biochar can also alter soil properties, such as pH, WHC_max_ and sorption capacities, which can affect the bioavailability of pharmaceuticals. The adsorption of biochar on pharmaceuticals and other organic micropollutants on biochar involves various mechanisms such as pore filling, physical interactions such as electrostatic forces, hydrogen bonding, hydrophobic effects, and π–π interactions.^65^ These interactions can be reversible, indicating that the adsorbed pharmaceuticals might be released back into the soil over time. Neverthless, according to study^66^ biochar can be physically, chemically, or biologically modified to alter its properties and enhance soil improvement, pollution remediation in order to maximize its benefits. Moreover, biochar use in terrestrial environments has its drawbacks. It can lead to the release of polycyclic aromatic hydrocarbons into the soil,^67^ and can prolong the degradation of pharmaceuticals.^19,65^

#### 3.2.3 Impact of Biochar on Degradation Rate of PhACs in Soil

The effect of biochar on the first-order degradation kinetics of PhACs in plant–soil-biochar system was found to be statistically significant (*p* < 0.05) in the case of 12 out of 25 PhACs, as shown in Table 1. Specifically, the addition of 2.0% biochar to soil increased the degradation rate of azithromycin (from *k* = 0.00076 d^–1^ to *k* = 0.0129 d^–1^), enrofloxacin (from *k* = 0.0092 d^–1^ to *k* = 0.0157 d^–1^) and sulfamethazine (from *k* = 0.052 d^–1^ to *k* = 0.125 d^–1^), but decreased the degradation rates of chlortetracycline (from *k* = 0.037 d^–1^ to *k* = 0.023 d^–1^), erythromycin (from *k* = 0.073 d^–1^ to *k* = 0.019 d^–1^), pefloxacin (from *k* = 0.116 d^–1^ to *k* = 0.0128 d^–1^), roxithromycin (from *k* = 0.098 d^–1^ to *k* = 0.023 d^–1^), sulfacetamide (from *k* = 0.198 d^–1^ to *k* = 0.113 d^–1^), sulfadiazine (from *k* = 0.0994 d^–1^ to *k* = 0.082 d^–1^), sulfamethoxypyridazine (from *k* = 0.074 d^–1^ to *k* = 0.055 d^–1^), sulfathiazole (from *k* = 0.133 d^–1^ to *k* = 0.090 d^–1^) and trimethoprim (from *k* = 0.022 d^–1^ to *k* = 0.094 d^–1^). A similar observation was made in a study,^19^ where the addition of 1.0% biochar to the soil increased the half-life of seven pharmaceuticals. The extent of this effect varied depending on the properties of both biochar and pharmaceuticals. The increased degradation time could be due to biochar ability to enhance soil adsorption, thereby lowering the proportion of pharmaceuticals accessible in the pore space, which bacteria rely on for degradation. This ultimately leads to a slower biodegradation rate.^68,69^ However, some studies^70,71^ have found that biochar can actually speed up degradation rates by providing a favorable environment for microbial colonization and activity, offering them carbon and other vital nutrients.

However, the increased persistence of pharmaceuticals in soils could counterbalance the benefits of reduced bioavailability, potentially leading to greater plant uptake and accumulation over time.^19^ This is consistent with our findings (as illustrated in Figures 1–3 and S1), where PhAC uptake by plants did not show a statistically significant difference between nonamended and biochar-amended soils.

### 3.3 Ecotoxicological End Points

#### 3.3.1 Concentration Range

**Figure 4:**
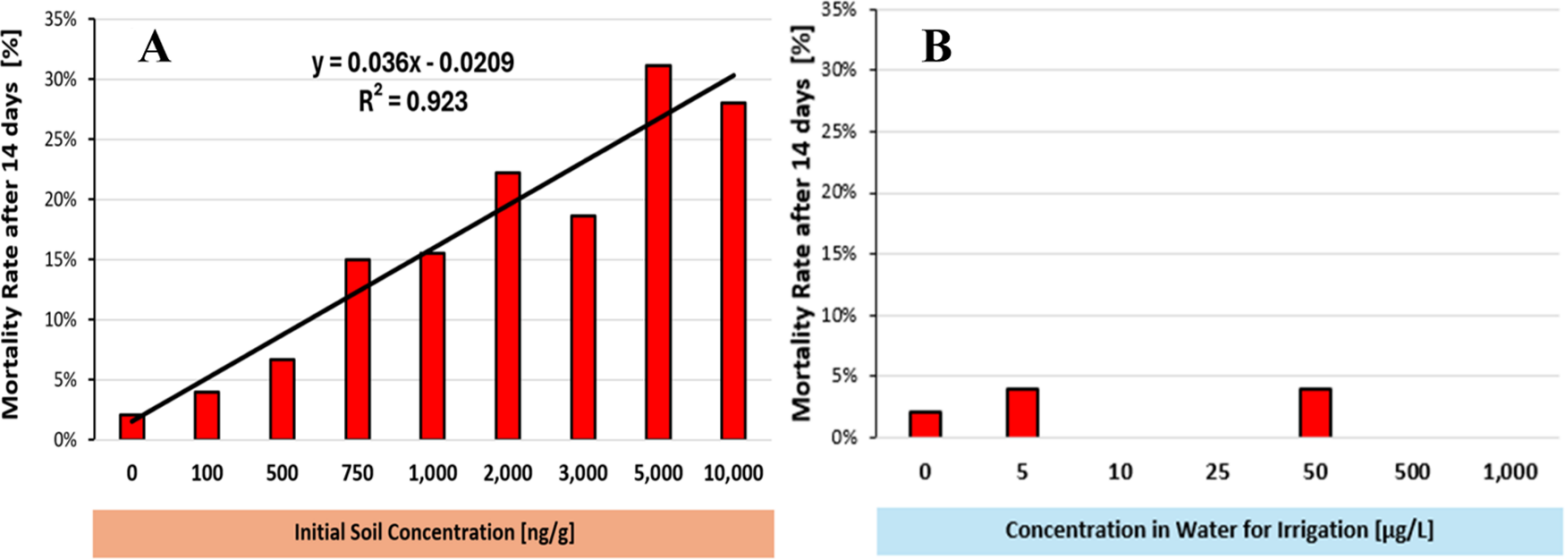
Mortality rate of lettuce seedlings after 14 days of exposure to pharmaceuticals; (A) Experiment with initially contaminated soil and (B) Experiment with contaminated water for irrigation.

In the experiment where *L. sativa* seedlings were grown in soil contaminated with various concentrations of a PhAC mixture, a significant increase in mortality was observed compared to the control group (Figure 4A). This mortality was directly related to the initial PhAC concentration in the soil and could be described using a linear regression model (*y* = 0.036*x* – 0.0209, *R*^2^ = 0.923). In contrast, when the seedlings were grown in uncontaminated soil but irrigated with water containing varying concentrations of PhACs, no significant differences in mortality were observed compared to the control group (Figure 4B). This suggests that soil contamination at the beginning of the experiment had a more significant effect on seedling mortality than contamination through irrigation. This disparity can be attributed to the lower initial PhAC concentrations observed at the beginning of the experiment when irrigated with contaminated water. Unlike direct soil contamination, where PhACs are present at higher concentrations from the outset, irrigation introduces PhACs gradually over time. High initial soil concentrations of contaminants can occur when animal manure is applied to agricultural fields.^72,73^

**Figure 5:**
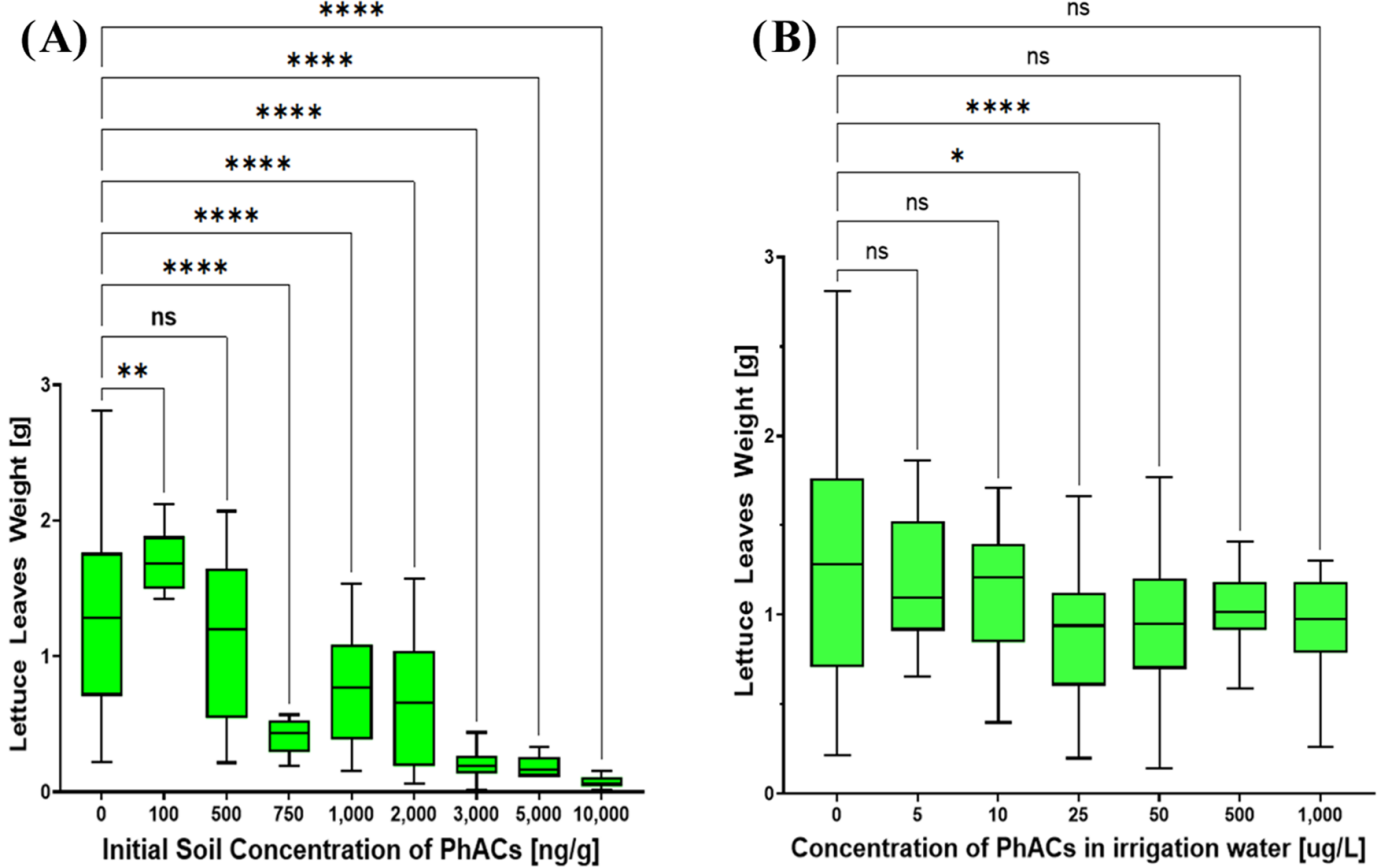
Lettuce leaves weight after 14 days of exposure to different treatments and concentrations of pharmaceutical mixture in soil, (A) Experiment with initially contaminated soil and (B) Experiment with contaminated water for irrigation. Significant effects are indicated by asterisks (*) or “ns” for nonsignificant results.

**Figure 6:**
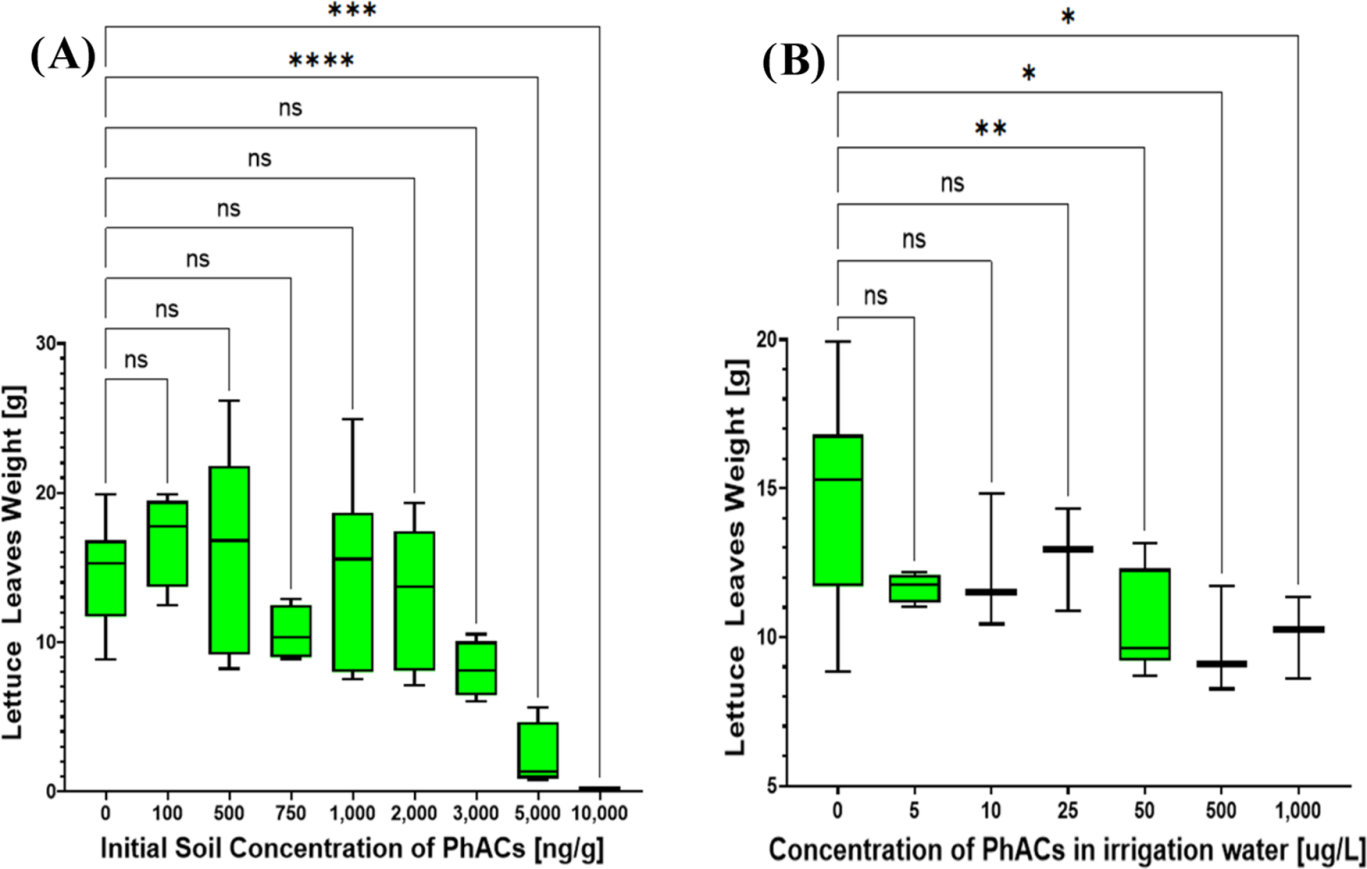
Lettuce leaves weight after 28 days of exposure to different treatments and concentrations of pharmaceutical mixture in soil, (A) Experiment with initially contaminated soil and (B) Experiment with contaminated water for irrigation. Significant effects are indicated by asterisks (*) or “ns” for nonsignificant results.

As an essential ecotoxicological end point, the weight of lettuce leaves was weighted after 14 and 28 days of growth in contaminated soil. To assess whether the concentration of a PhAC mixture in the soil significantly affected the above-ground biomass compared to the control group, Dunnett’s test was employed, with statistically significant effects indicated by asterisks in Figures 5–6.

After 14 days, consistent with the higher mortality rates, the above-ground biomass was more impacted in the group with initially contaminated soil (Figure 5A) compared to the group irrigated with contaminated water (Figure 5B). When the soil was initially contaminated with a concentration of 100 ng·g^–1^, an increase in biomass weight indicative of hormesis was observed (*p* < 0.05), consistent with previous studies.^28,74,75^ However, at a higher of concentration of 500 ng·g^–1^, no significant effect on biomass weight was observed (*p* > 0.05). Moreover, decreased biomass weight was observed at PhAC concentrations ranging from 750 to 10,000 ng·g^–1^ (*p* < 0.05), with the impact varying according to the specific PhAC concentrations. In agreement with a previous study,^74^ hormesis was observed at low doses of xenobiotics and displayed a nonlinear dose–response relationship. Futhermore, in the experiment where lettuces were irrigated with water containing PhACs (Figure 5B), significant effects on biomass weight were observed only at concentrations of 25 and 50 μg·L^–1^ (*p* < 0.05). The effect of water irrigation concentrations on biomass was inconsistent, as neither lower nor higher levels of irrigation resulted in statistically significant changes (*p* > 0.05). A closer examination of the boxplots (Figure 5B), which display the interquartile range (Q1-Q3) and median values across the pharmaceutical concentrations, reveals minimal differences among the various treatments. These minimal differences suggest that, while the 25 and 50 μg·L^–1^ concentrations are statistically significant, the actual impact on biomass is not substantial compared to other concentrations. The statistically significant differences observed at 25 and 50 μg·L^–1^ may be attributed to several factors. First, slight variations in phytotoxicity could result in minor reductions in biomass that become statistically significant. Second, the biological variability of *L. sativa* might contribute to these differences. Third, potential differences in the bioavailability of PhACs at these concentrations could play a role. Lastly, experimental variability, such as slight inconsistencies in the experimental conditions, could also contribute to the observed significance. Therefore, these factors combined might lead to statistically significant results, despite the minimal practical differences in biomass observed.

After 28 days, the effect of the PhAC mixture on lettuce biomass was reduced at lower soil concentrations (Figure 6A). This mitigation could be attributed to lettuce growth, degradation of PhACs in the soil (as suggested in Table 1 and Figures 1–3), or a combination of both factors. Despite the decreasing concentrations of parent drugs, various PhAC metabolites are formed, which often remain unidentified and unquantified in available studies.^76,77^ Notably, significant effects on biomass were observed only at initial soil concentrations above 5000 ng·g^–1^ (*p* < 0.05, Figure 6A). In contrast, when the lettuces were irrigated with water containing PhACs, significant phytotoxicity was observed only at concentrations above 50 μg·L^–1^ (*p* < 0.05, Figure 6B).

These findings align with a study,^78^ in which lettuces were irrigated with a mixture of 14 PhACs at concentrations of 10 and 100 μg·L^–1^ in both water and wastewater. While no significant effects on lettuce biomass were observed at these concentrations, the same study^78^ reported significant changes in the structure of the soil bacterial community. Additionally, our results align with a study,^75^ which found that environmentally relevant concentrations of antibiotics (i.e., ≤360 ng·g^–1^ in soil) did not adversely affect the growth and yields of radishes, lettuce, and fescue grass (*Festuca arundinaceaSchreb.*).

Additionaly, PhACs can negatively affect plant seed germination, with effects varying by plant species and the type of antibiotics used. A study^30^ reported significant reductions in germination rates with colistin (up to 89%), amoxicillin (up to 64%), and ampicillin (100%). However, contrasting findings were reported in another study,^31^ where no significant effects on seed germination were observed in a soil environment contaminated with a range of 0.01–10,000 ng·g^–1^ of individual PhACs, including paracetamol, ibuprofen, and amoxicillin. These discrepancies could be explained by the protective nature of the seed coat, which can protect the plant embryo from adverse environmental conditions, as supported by previous studies.^32,33^ This inherent resilience of seeds may lead to variability in the observed effects of PhACs on germination. Furthermore, when dealing with PhAC mixtures, which more accurately represent real-world environmental contamination compared to single-compound exposure, there is the potential for additive or antagonistic toxicity.^25^ Additionally, conducting exposure experiments with individual substances is nearly impossible because of their high quantity and increasing number.

Overall, phytotoxicity caused by PhACs can manifest in various ways, including reduced germination rates, chlorosis, tissue deformation, shortened or diminished root and shoot mass, decreased reproductive rates, oxidative stress, and altered enzymatic activity. The specific symptoms depend on factors such as contamination concentrations, the type of pharmaceutical involved, and its therapeutic class.^28,31^

Moreover, it has been suggested that plant hormone homeostasis could be disrupted by PhACs at much lower concentrations than those typically required to affect biomass visibly.^74^ This indicates that even low levels of PhACs in the environment may have a more profound impact on plant physiological processes than initially expected.

#### 3.3.2 Biochar Effect

**Figure 7:**
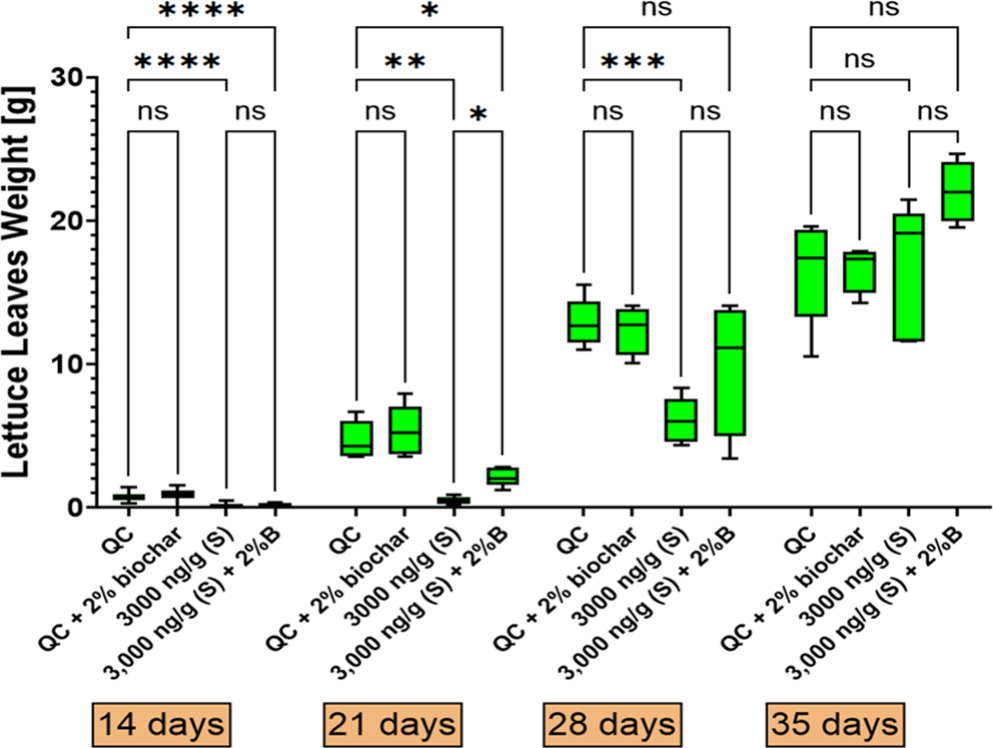
Lettuce leaves weight during exposure to pharmaceutical mixture in nonamended and biochar-amended soil. Significant effects are indicated by asterisks (*) or “ns” for nonsignificant results.

To assess whether biochar could mitigate the effects of PhAC contamination, additional experiments were conducted using both nonamended soil and biochar-amended soil. These experiments included a control group without PhAC contamination and a soil group with a concentration of 3000 ng·g^–1^. Regarding mortality rates, biochar did not significantly affect the outcome. To further evaluate the impact on plant growth, the aboveground biomass weights were measured at days 14, 21, 28, and 35 (Figure 7, with statistically significant effects marked by asterisks). At the beginning of the experiment, the presence of a mixture of pharmaceuticals in the soil significantly reduced the biomass weight of lettuce (*p* < 0.05). However, by day 35, this effect had largely diminished (*p* > 0.05, Figure 7), likely due to the degradation of PhACs in the soil (Figures 1–3), which allowed lettuce to reach the recovery stage. The application of biochar had a partially significant effect on lettuce weight. When comparing noncontaminated and contaminated soils, significant differences were observed up to day 35. However, with the addition of biochar to PhAC-contaminated soil, significant differences were only observed up to day 28. Additionally, a significant difference (*p* < 0.05, Figure 7) was observed between PhAC-contaminated soil with and without biochar amendment on day 21. These findings suggest that biochar application can partially mitigate the phytotoxicity effect of PhAC contamination over time, allowing *L. sativa* to recover more quickly. Moreover, although no significant difference (*p* > 0.05) was observed on day 35 between the noncontaminated and PhAC-contaminated soil amended with 2% biochar, the box plot (Figure 7) suggests a possible hormetic effect. This could mean that a low level of stress from PhACs, in combination with biochar, might stimulate growth, suggesting a complex relationship between contaminants, biochar, and plant responses.

Similarly to our results, a study^54^ found that biochar amendment can reduce phytotoxicity by decreasing the bioavailability of organic pollutants. Another study^34^ concluded that the addition of biochar can boost both root growth and shoot biomass, with statistically significant improvements (*p* < 0.05). However, despite the positive effects of biochar, it can also have negative impacts, such as releasing heavy metals or polycyclic aromatic hydrocarbons, and altering nutrient bioavailability.^54^

### 3.4 Potential Health Risk and Risk toward Antimicrobial Resistance

To evaluate whether pharmaceutical residues in 28-day old lettuce pose a potential health risk, we estimated the hazard index (HI) as the sum of risk quotients (RQs) for each pharmaceutical. The calculation method is detailed in the Supporting Information, Appendix 3, and is based on the approaches used in previous studies.^36,37^ Hazard indexes were determined for different soil treatments, including direct soil spiking and irrigation with contaminated water, at two concentration levels (soil spike at 100 and 1000 ng·g^–1^, soil irrigation at 5 and 50 μg·L^–1^), simulating a range of environmentally relevant concentrations (Table S6).

Consistent with other studies,^8,17,34^ the estimated HI values did not exceed the thresholds of 0.01 or 0.05, which are considered benchmarks for considerable and distinct human risk, respectively.^37^ In the study,^8^ lettuce and spinach were hydroponically grown in nutrient solutions containing 20 pharmaceutical and personal care products (PPCPs) at concentrations of 0.5 or 5 μg·L^–1^. Despite the generally higher uptake rates in hydroponic systems compared to soil, the study found the associated health risks to be negligible.^8^ In study,^17^ carrots and lettuce were grown in uncontaminated soil and irrigated with water containing tetracycline and amoxicillin at concentrations of 0.1, 1.0, 10.0, or 15.0 mg·L^–1^. The study found that estimated daily intakes (EDIs) for these antibiotics were several thousand times lower than their acceptable daily intakes (ADIs), suggesting that consumer exposure through plants is likely well below the ADI and the risk of toxicity is probably low. However, the study highlights that antibiotic resistance is the primary health concern.^17^ Similarly, in study,^34^ lettuce and carrots were grown in soil with ciprofloxacin (100 mg·kg^–1^), triclocarban (500 mg·kg^–1^), and triclosan (200 mg·kg^–1^). The ADI values for these compounds were much higher than the exposure estimates from plant biomass. While not directly toxic, these pharmaceuticals may pose risks such as endocrine disruption, antibiotic resistance, or long-term health effects, highlighting the need for further research.^34^

These studies and our results (Table S6) suggest that, the health risk from pharmaceutical residues in vegetable (including lettuce) is negligible.^8,17^ Although, it is important to recognize that while we have calculated HI for pharmaceutical intake through lettuce, lettuce is just one type of commonly consumed vegetable. Other vegetables could be contaminated at similar levels due to factors like wastewater irrigation or the application of animal manure or biosolids to agricultural land. This indicates that the potential risk from pharmaceuticals may extend beyond a single food source, underscoring the need for a broader assessment of food safety in the context of pharmaceutical contamination.

Furthermore, it is important to mention that vegetable consumption varies widely across the population, and our analysis used only the average value, without accounting for worst-case exposure scenarios such as those involving vegetarian or vegan diets, where an individual might consume up to 500 g of vegetables twice a day.^79^ Moreover, in real-world settings, soil or wastewater contamination could involve a broader range of pharmaceuticals and other micropollutants, along with their metabolites and degradation products, which are often unknown. This could lead to an underestimation of human exposure.^36,80^ Humans are also likely to be exposed to micropollutants from various sources, including food crops, drinking water, and home and workplace environments.^81^ Additionally, one of the most significant concerns associated with pharmaceuticals in the environment is the rise of antimicrobial resistance.^17,34,37,82^ Moreover, a study^83^ demonstrated the horizontal transfer of resistance genes from crops to the gut microbiome of mice, highlighting the potential risks of gene transfer due to the presence of resistance genes in contaminated vegetables. This emphasizes the need for careful monitoring and assessment of pharmaceutical contaminants in agriculture and their broader implications for public health.

Therefore, we also evaluated the risk quotients (RQs) for the emergence of antimicrobial resistance (AMR) in the soil environment at day 0 and after 28 days for various soil treatments and concentrations. Specifically, we considered soil spiked at concentrations of 100 and 1000 ng·g^–1^ and soil irrigation at concentrations of 5 and 50 μg·L^–1^, representing a range of environmentally relevant concentrations. RQ values were calculated as the ratio of the measured environmental concentration (MEC) to a predicted no-effect concentration (PNEC). Details of these calculations can be found in Supporting Information, Appendix 4, and the calculated RQs and ∑RQs are provided in Table S7. The criteria for interpreting RQs followed commonly used thresholds: low risk when RQ < 0.1, medium risk when 0.1 < RQ < 1, and high risk for RQ > 1.^84,85^Table S7 illustrates that both high risk (RQ > 1) and medium risk (RQ > 0.1) scenarios were observed, regardless of the soil contamination route (initially spiked soil versus irrigated soil). The primary difference in RQs stemmed from the total amount of pharmaceuticals introduced into the soil system. However, it is important to note that RQs gradually decreased over time due to the degradation of antibiotics in the soil environment. Nevertheless, the formation of degradation products or metabolites, for which RQs were not calculated, may have similar toxicity and contribute to the rise of AMR. These findings align with previous studies,^86,87^ underscoring the significant environmental and health concerns associated with the emergence and spread of AMR across environmental, animal, and human populations.

## 4 Conclusions

Within this study, we determined bioconcentration factors (BCF) and translocation factors (TF) for 25 pharmaceuticals, especially antibiotics commonly found in agricultural fields. For the calculation of BCFs and TFs, a novel approach using time-weighted average (TWA) soil concentrations was used, considering initial soil contamination and pharmaceutical degradation. Moreover, we evaluated the effectiveness of biochar in reducing the bioavailability of these compounds and conducted ecotoxicological tests. Surprisingly, we observed no significant impact on the bioavailability to *L. sativa*. However, the degradation kinetics of several pharmaceuticals were unintentionally prolonged, alongside partially positive results indicating the mitigation of phytotoxicity toward the biomass of *L. sativa*.

In addition, we calculated the estimated daily intake (EDI) of residues in vegetables, suggesting negligible health risks if only *L. sativa* leaves were contaminated. However, under real conditions, it is likely that other vegetables would be similarly contaminated. Because of potential additive effects, health risks could be present due to the intake of low concentrations of dozens of micropollutants. Lastly, we evaluated the potential environmental risk of antimicrobial resistance (AMR) in soil, where both risk quotients (RQs) and cumulative risk quotients (∑RQs) indicate significant concerns regarding the prevalence and spread of AMR across environmental, animal, and human populations.

## Data Availability

The data that support the findings of this study are available from the corresponding author, upon reasonable request.

## Supporting Information Available

Detailed tables and appendices including physicochemical properties of pharmaceuticals, soil, and biochar; extraction and LC-MS/MS methods and MRM transitions; health risk estimation and antimicrobial resistance risk quotients; and a heatmap of pharmaceutical distribution in lettuce samples (PDF)

## References

[ref1] Khasawneh O. F. S.; Palaniandy P. Occurrence and Removal of Pharmaceuticals in Wastewater Treatment Plants. Process Saf. Environ. Prot. 2021, 150, 532–556. 10.1016/j.psep.2021.04.045.

[ref2] Bijlsma L.; Pitarch E.; Fonseca E.; Ibáñez M.; Botero A. M.; Claros J.; Pastor L.; Hernández F. Investigation of Pharmaceuticals in a Conventional Wastewater Treatment Plant: Removal Efficiency, Seasonal Variation and Impact of a Nearby Hospital. J. Environ. Chem. Eng. 2021, 9 (4), 105548 10.1016/j.jece.2021.105548.

[ref3] Xia K.; Bhandari A.; Das K.; Pillar G. Occurrence and Fate of Pharmaceuticals and Personal Care Products (PPCPs) in Biosolids. J. Environ. Qual. 2005, 34 (1), 91–104. 10.2134/jeq2005.0091. 15647538

[ref4] Kinney C. A.; Heuvel B. V. Translocation of Pharmaceuticals and Personal Care Products after Land Application of Biosolids. Curr. Opin. Environ. Sci. Health 2020, 14, 23–30. 10.1016/j.coesh.2019.11.004.

[ref5] Li Y.; Sallach J. B.; Zhang W.; Boyd S. A.; Li H. Characterization of Plant Accumulation of Pharmaceuticals from Soils with Their Concentration in Soil Pore Water. Environ. Sci. Technol. 2022, 56 (13), 9346–9355. 10.1021/acs.est.2c00303. 35738923

[ref6] Kaczala F.; Blum S. E. The Occurrence of Veterinary Pharmaceuticals in the Environment: A Review. Curr. Anal. Chem. 2016, 12 (3), 169–182. 10.2174/1573411012666151009193108. 28579931 PMC5425647

[ref7] Białk-Bielińska A.; Kumirska J.; Borecka M.; Caban M.; Paszkiewicz M.; Pazdro K.; Stepnowski P. Selected Analytical Challenges in the Determination of Pharmaceuticals in Drinking/Marine Waters and Soil/Sediment Samples. J. Pharm. Biomed. Anal. 2016, 121, 271–296. 10.1016/j.jpba.2016.01.016. 26818066

[ref8] Wu X.; Ernst F.; Conkle J. L.; Gan J. Comparative Uptake and Translocation of Pharmaceutical and Personal Care Products (PPCPs) by Common Vegetables. Environ. Int. 2013, 60, 15–22. 10.1016/j.envint.2013.07.015. 23973619

[ref9] Mordechay E. B.; Mordehay V.; Tarchitzky J.; Chefetz B. Fate of Contaminants of Emerging Concern in the Reclaimed Wastewater-Soil-Plant Continuum. Sci. Total Environ. 2022, 822, 153574 10.1016/j.scitotenv.2022.153574. 35114239

[ref10] Riva F.; Zuccato E.; Pacciani C.; Colombo A.; Castiglioni S. A Multi-Residue Analytical Method for Extraction and Analysis of Pharmaceuticals and Other Selected Emerging Contaminants in Sewage Sludge. Anal. Methods 2021, 13 (4), 526–535. 10.1039/D0AY02027C. 33447838

[ref11] Mordechay E. B.; Tarchitzky J.; Chen Y.; Shenker M.; Chefetz B. Composted Biosolids and Treated Wastewater as Sources of Pharmaceuticals and Personal Care Products for Plant Uptake: A Case Study with Carbamazepine. Environ. Pollut. 2018, 232, 164–172. 10.1016/j.envpol.2017.09.029. 28935405

[ref12] Kodešová R.; Klement A.; Golovko O.; Fér M.; Kočárek M.; Nikodem A.; Grabic R. Soil Influences on Uptake and Transfer of Pharmaceuticals from Sewage Sludge Amended Soils to Spinach. J. Environ. Manage. 2019, 250, 109407 10.1016/j.jenvman.2019.109407. 31472377

[ref13] Mejías C.; Martín J.; Santos J. L.; Aparicio I.; Alonso E. Occurrence of Pharmaceuticals and Their Metabolites in Sewage Sludge and Soil: A Review on Their Distribution and Environmental Risk Assessment. Trends Environ. Anal. Chem. 2021, 30, e00125 10.1016/j.teac.2021.e00125.

[ref14] Gros M.; Mas-Pla J.; Boy-Roura M.; Geli I.; Domingo F.; Petrović M. Veterinary Pharmaceuticals and Antibiotics in Manure and Slurry and Their Fate in Amended Agricultural Soils: Findings from an Experimental Field Site (Baix Empordà, NE Catalonia). Sci. Total Environ. 2019, 654, 1337–1349. 10.1016/j.scitotenv.2018.11.061. 30841406

[ref15] Gworek B.; Kijeńska M.; Wrzosek J.; Graniewska M. Pharmaceuticals in the Soil and Plant Environment: A Review. Water, Air, Soil Pollut. 2021, 232 (4), 145 10.1007/s11270-020-04954-8.

[ref16] Santiago S.; Roll D. M.; Ray C.; Williams C.; Moravcik P.; Knopf A. Effects of Soil Moisture Depletion on Vegetable Crop Uptake of Pharmaceuticals and Personal Care Products (PPCPs). Environ. Sci. Pollut. Res. 2016, 23 (20), 20257–20268. 10.1007/s11356-016-7194-z. 27447471

[ref17] Azanu D.; Mortey C.; Darko G.; Weisser J. J.; Styrishave B.; Abaidoo R. C. Uptake of Antibiotics from Irrigation Water by Plants. Chemosphere 2016, 157, 107–114. 10.1016/j.chemosphere.2016.05.035. 27213239

[ref18] Sallach J. B.; Zhang Y.; Hodges L.; Snow D.; Li X.; Bartelt-Hunt S. Concomitant Uptake of Antimicrobials and Salmonella in Soil and into Lettuce Following Wastewater Irrigation. Environ. Pollut. 2015, 197, 269–277. 10.1016/j.envpol.2014.11.018. 25483595

[ref19] Li Y.; He J.; Qi H.; Li H.; Boyd S. A.; Zhang W. Impact of Biochar Amendment on the Uptake, Fate and Bioavailability of Pharmaceuticals in Soil-Radish Systems. J. Hazard. Mater. 2020, 398, 122852 10.1016/j.jhazmat.2020.122852. 32512441

[ref20] Stando K.; Korzeniewska E.; Felis E.; Harnisz M.; Bajkacz S. Uptake of Pharmaceutical Pollutants and Their Metabolites from Soil Fertilized with Manure to Parsley Tissues. Molecules 2022, 27 (14), 4378 10.3390/molecules27144378. 35889250 PMC9317704

[ref21] Malchi T.; Maor Y.; Tadmor G.; Shenker M.; Chefetz B. Irrigation of Root Vegetables with Treated Wastewater: Evaluating Uptake of Pharmaceuticals and the Associated Human Health Risks. Environ. Sci. Technol. 2014, 48 (16), 9325–9333. 10.1021/es5017894. 25026038

[ref22] Prosser R. S.; Trapp S.; Sibley P. K. Modeling Uptake of Selected Pharmaceuticals and Personal Care Products into Food Crops from Biosolids-Amended Soil. Environ. Sci. Technol. 2014, 48 (19), 11397–11404. 10.1021/es503067v. 25207852

[ref23] Sabourin L.; Duenk P.; Bonte-Gelok S.; Payne M.; Lapen D. R.; Topp E. Uptake of Pharmaceuticals, Hormones and Parabens into Vegetables Grown in Soil Fertilized with Municipal Biosolids. Sci. Total Environ. 2012, 431, 233–236. 10.1016/j.scitotenv.2012.05.017. 22687432

[ref24] Riemenschneider C.; Al-Raggad M.; Moeder M.; Seiwert B.; Salameh E.; Reemtsma T. Pharmaceuticals, Their Metabolites, and Other Polar Pollutants in Field-Grown Vegetables Irrigated with Treated Municipal Wastewater. J. Agric. Food Chem. 2016, 64 (29), 5784–5792. 10.1021/acs.jafc.6b01696. 27378214

[ref25] Bhalsod G. D.; Chuang Y.-H.; Jeon S.; Gui W.; Li H.; Ryser E. T.; Guber A. K.; Zhang W. Uptake and Accumulation of Pharmaceuticals in Overhead- and Surface-Irrigated Greenhouse Lettuce. J. Agric. Food Chem. 2018, 66 (4), 822–830. 10.1021/acs.jafc.7b04355. 29293328

[ref26] Bassil R. J.; Bashour I. I.; Sleiman F. T.; Abou-Jawdeh Y. A. Antibiotic Uptake by Plants from Manure-Amended Soils. J. Environ. Sci. Health, Part B 2013, 48 (7), 570–574. 10.1080/03601234.2013.774898. 23581689

[ref27] Chuang Y.-H.; Liu C.-H.; Sallach J. B.; Hammerschmidt R.; Zhang W.; Boyd S. A.; Li H. Mechanistic Study on Uptake and Transport of Pharmaceuticals in Lettuce from Water. Environ. Int. 2019, 131, 104976 10.1016/j.envint.2019.104976. 31336255

[ref28] Akenga P.; Gachanja A.; Fitzsimons M. F.; Tappin A.; Comber S. Uptake, Accumulation and Impact of Antiretroviral and Antiviral Pharmaceutical Compounds in Lettuce. Sci. Total Environ. 2021, 766, 144499 10.1016/j.scitotenv.2020.144499. 33418261

[ref29] Yu X.; Liu X.; Liu H.; Chen J.; Sun Y. The Accumulation and Distribution of Five Antibiotics from Soil in 12 Cultivars of Pak Choi. Environ. Pollut. 2019, 254, 113115 10.1016/j.envpol.2019.113115. 31476671

[ref30] Benassi-Borba L.; Dal’Lin C. M. P.; Testolin R. C.; Vieira N. M. B.; Corrêa C. V. T.; Bianchi I.; Barwinski M. J. B.; Radetski C. M.; Somensi C. A. Assessment of Phytotoxicity and Impact on the Enzymatic Activity of Soil Microorganisms Caused by Veterinary Antibiotics Used in Brazilian Farms. J. Environ. Sci. Health, Part B 2021, 56 (7), 675–684. 10.1080/03601234.2021.1938480. 34319219

[ref31] Rede D.; Santos L. H. M. L. M.; Ramos S.; Oliva-Teles F.; Antão C.; Sousa S. R.; Delerue-Matos C. Individual and Mixture Toxicity Evaluation of Three Pharmaceuticals to the Germination and Growth of *Lactuca Sativa* Seeds. Sci. Total Environ. 2019, 673, 102–109. 10.1016/j.scitotenv.2019.03.432. 30986672

[ref32] Hillis D. G.; Fletcher J.; Solomon K. R.; Sibley P. K. Effects of Ten Antibiotics on Seed Germination and Root Elongation in Three Plant Species. Arch. Environ. Contam. Toxicol. 2011, 60 (2), 220–232. 10.1007/s00244-010-9624-0. 21107831

[ref33] Pino M. R.; Muñiz S.; Val J.; Navarro E. Phytotoxicity of 15 Common Pharmaceuticals on the Germination of *Lactuca Sativa* and Photosynthesis of *Chlamydomonas Reinhardtii*. Environ. Sci. Pollut. Res. 2016, 23 (22), 22530–22541. 10.1007/s11356-016-7446-y. 27553001

[ref34] Bair D. A.; Anderson C. G.; Chung Y.; Scow K. M.; Franco R. B.; Parikh S. J. Impact of Biochar on Plant Growth and Uptake of Ciprofloxacin, Triclocarban and Triclosan from Biosolids. J. Environ. Sci. Health, Part B 2020, 55 (11), 990–1001. 10.1080/03601234.2020.1807264. 32877275

[ref35] Keerthanan S.; Jayasinghe C.; Bolan N.; Rinklebe J.; Vithanage M. Retention of Sulfamethoxazole by Cinnamon Wood Biochar and Its Efficacy of Reducing Bioavailability and Plant Uptake in Soil. Chemosphere 2022, 297, 134073 10.1016/j.chemosphere.2022.134073. 35227748

[ref36] Geng J.; Liu X.; Wang J.; Li S. Accumulation and Risk Assessment of Antibiotics in Edible Plants Grown in Contaminated Farmlands: A Review. Sci. Total Environ. 2022, 853, 158616 10.1016/j.scitotenv.2022.158616. 36089029

[ref37] Keerthanan S.; Jayasinghe C.; Biswas J. K.; Vithanage M. Pharmaceutical and Personal Care Products (PPCPs) in the Environment: Plant Uptake, Translocation, Bioaccumulation, and Human Health Risks. Crit. Rev. Environ. Sci. Technol. 2021, 51 (12), 1221–1258. 10.1080/10643389.2020.1753634.

[ref38] Mieczyslaw G.; Janas R. Physiological method for improving seed germination and seedling emergence of root parsley in organic systems. J. Res. Appl. Agric. Eng. 2014, 59, 80–86.

[ref39] Bożym M. Assessment of Phytotoxicity of Leachates from Landfilled Waste and Dust from Foundry. Ecotoxicology 2020, 29 (4), 429–443. 10.1007/s10646-020-02197-1. 32291613 PMC7182548

[ref40] Holatko J.; Brtnicky M.; Mustafa A.; Kintl A.; Skarpa P.; Ryant P.; Baltazar T.; Malicek O.; Latal O.; Hammerschmiedt T. Effect of Digestate Modified with Amendments on Soil Health and Plant Biomass under Varying Experimental Durations. Materials 2023, 16 (3), 1027 10.3390/ma16031027. 36770034 PMC9920836

[ref41] Mravcová L.; Amrichová A.; Navrkalová J.; Hamplová M.; Sedlář M.; Gargošová H. Z.; Fučík J. Optimization and Validation of Multiresidual Extraction Methods for Pharmaceuticals in Soil, Lettuce, and Earthworms. Environ. Sci. Pollut. Res. 2024, 31, 33120–33140. 10.1007/s11356-024-33492-7. PMC11133184 38676866

[ref42] Pang Z.; Lu Y.; Zhou G.; Hui F.; Xu L.; Viau C.; Spigelman A. F.; MacDonald P. E.; Wishart D. S.; Li S.; Xia J. MetaboAnalyst 6.0: Towards a Unified Platform for Metabolomics Data Processing, Analysis and Interpretation. Nucleic Acids Res. 2024, 52, gkae253 10.1093/nar/gkae253. PMC11223798 38587201

[ref43] Bao Y.; Li Y.; Pan C. Effects of the Removal of Soil Extractable Oxytetracycline Fractions on Its Bioaccumulation in Earthworm and Horsebean. Water, Air, Soil Pollut. 2018, 229 (3), 79 10.1007/s11270-018-3742-0.

[ref44] Wu X.; Dodgen L. K.; Conkle J. L.; Gan J. Plant Uptake of Pharmaceutical and Personal Care Products from Recycled Water and Biosolids: A Review. Sci. Total Environ. 2015, 536, 655–666. 10.1016/j.scitotenv.2015.07.129. 26254067

[ref45] Schmidt T.; Kimmel S.; Hoeger S.; Lemic D.; Bazok R.; Gasparic H. V. Plant Protection Products in Agricultural Fields – Residues in Earthworms and Assessment of Potentially Toxic Effects to the Environment. J. Cent. Eur. Agric. 2022, 23 (3), 604–614. 10.5513/JCEA01/23.3.3625.

[ref46] Ma X.; Zhang H.; Wang Z.; Yao Z.; Chen J.; Chen J. Bioaccumulation and Trophic Transfer of Short Chain Chlorinated Paraffins in a Marine Food Web from Liaodong Bay, North China. Environ. Sci. Technol. 2014, 48 (10), 5964–5971. 10.1021/es500940p. 24745704

[ref47] Zhu M.; Chen J.; Peijnenburg W. J. G. M.; Xie H.; Wang Z.; Zhang S. Controlling Factors and Toxicokinetic Modeling of Antibiotics Bioaccumulation in Aquatic Organisms: A Review. Crit. Rev. Environ. Sci. Technol. 2023, 53 (15), 1431–1451. 10.1080/10643389.2022.2142033.

[ref48] Hyland K. C.; Blaine A. C.; Higgins C. P. Accumulation of Contaminants of Emerging Concern in Food Crops—Part 2: Plant Distribution. Environ. Toxicol. Chem. 2015, 34 (10), 2222–2230. 10.1002/etc.3068. 25988579

[ref49] Knight E. R.; Carter L. J.; McLaughlin M. J. Bioaccumulation, Uptake, and Toxicity of Carbamazepine in Soil–Plant Systems. Environ. Toxicol. Chem. 2018, 37 (4), 1122–1130. 10.1002/etc.4053. 29193285

[ref50] Conde-Cid M.; Ferreira-Coelho G.; Fernández-Calviño D.; Núñez-Delgado A.; Fernández-Sanjurjo M. J.; Arias-Estévez M.; Álvarez-Rodríguez E. Single and Simultaneous Adsorption of Three Sulfonamides in Agricultural Soils: Effects of PH and Organic Matter Content. Sci. Total Environ. 2020, 744, 140872 10.1016/j.scitotenv.2020.140872. 32711315

[ref51] Al-Farsi R. S.; Ahmed M.; Al-Busaidi A.; Choudri B. S. Translocation of Pharmaceuticals and Personal Care Products (PPCPs) into Plant Tissues: A Review. Emerging Contam. 2017, 3 (4), 132–137. 10.1016/j.emcon.2018.02.001.

[ref52] Al-Farsi R.; Ahmed M.; Al-Busaidi A.; Choudri B. S. Assessing the Presence of Pharmaceuticals in Soil and Plants Irrigated with Treated Wastewater in Oman. Int. J. Recycl. Org. Waste Agric. 2018, 7 (2), 165–172. 10.1007/s40093-018-0202-1.

[ref53] Hyland K. C.; Blaine A. C.; Dickenson E. R. V.; Higgins C. P. Accumulation of Contaminants of Emerging Concern in Food Crops—Part 1: Edible Strawberries and Lettuce Grown in Reclaimed Water. Environ. Toxicol. Chem. 2015, 34 (10), 2213–2221. 10.1002/etc.3066. 25988333

[ref54] Caban M.; Folentarska A.; Lis H.; Kobylis P.; Bielicka-Giełdoń A.; Kumirska J.; Ciesielski W.; Stepnowski P. Critical Study of Crop-Derived Biochars for Soil Amendment and Pharmaceutical Ecotoxicity Reduction. Chemosphere 2020, 248, 125976 10.1016/j.chemosphere.2020.125976. 32006830

[ref55] Chung H. S.; Lee Y.-J.; Rahman Md. M.; Abd El-Aty A. M.; Lee H. S.; Kabir Md. H.; Kim S. W.; Park B.-J.; Kim J.-E.; Hacımüftüoğlu F.; Nahar N.; Shin H.-C.; Shim J.-H. Uptake of the Veterinary Antibiotics Chlortetracycline, Enrofloxacin, and Sulphathiazole from Soil by Radish. Sci. Total Environ. 2017, 605–606, 322–331. 10.1016/j.scitotenv.2017.06.231. 28668743

[ref56] Tasho R. P.; Cho J. Y. Veterinary Antibiotics in Animal Waste, Its Distribution in Soil and Uptake by Plants: A Review. Sci. Total Environ. 2016, 563–564, 366–376. 10.1016/j.scitotenv.2016.04.140. 27139307

[ref57] Chowdhury K. F.; Hall R. J.; McNally A.; Carter L. J. Phytoremediation as a Tool to Remove Drivers of Antimicrobial Resistance in the Aquatic Environment. Rev. Environ. Contam. Toxicol. 2023, 261 (1), 16 10.1007/s44169-023-00039-9.

[ref58] Singh S.; Pant A.; Dutta K.; Rani R.; Vithanage M.; Daverey A. Phytoremediation of Pharmaceuticals and Personal Care Products Using the Constructed Wetland. Environ. Chem. Ecotoxicol. 2024, 6, 104–116. 10.1016/j.enceco.2024.04.001.

[ref59] Li Y.; Lian J.; Wu B.; Zou H.; Tan S. K. Phytoremediation of Pharmaceutical-Contaminated Wastewater: Insights into Rhizobacterial Dynamics Related to Pollutant Degradation Mechanisms during Plant Life Cycle. Chemosphere 2020, 253, 126681 10.1016/j.chemosphere.2020.126681. 32278919

[ref60] Carter L. J.; Williams M.; Martin S.; Kamaludeen S. P. B.; Kookana R. S. Sorption, Plant Uptake and Metabolism of Benzodiazepines. Sci. Total Environ. 2018, 628–629, 18–25. 10.1016/j.scitotenv.2018.01.337. 29428856

[ref61] Paz A.; Tadmor G.; Malchi T.; Blotevogel J.; Borch T.; Polubesova T.; Chefetz B. Fate of Carbamazepine, Its Metabolites, and Lamotrigine in Soils Irrigated with Reclaimed Wastewater: Sorption, Leaching and Plant Uptake. Chemosphere 2016, 160, 22–29. 10.1016/j.chemosphere.2016.06.048. 27351902

[ref62] Chitara M. K.; Chauhan S.; Singh R. P. Bioremediation of Polluted Soil by Using Plant Growth–Promoting Rhizobacteria. In Microorganisms for Sustainability; Springer, 2021; pp 203–226.

[ref63] Shen X.; Dai M.; Yang J.; Sun L.; Tan X.; Peng C.; Ali I.; Naz I. A Critical Review on the Phytoremediation of Heavy Metals from Environment: Performance and Challenges. Chemosphere 2022, 291, 132979 10.1016/j.chemosphere.2021.132979. 34801572

[ref64] Bhat S. A.; Bashir O.; Ul Haq S. A.; Amin T.; Rafiq A.; Ali M.; Américo-Pinheiro J. H. P.; Sher F. Phytoremediation of Heavy Metals in Soil and Water: An Eco-Friendly, Sustainable and Multidisciplinary Approach. Chemosphere 2022, 303, 134788 10.1016/j.chemosphere.2022.134788. 35504464

[ref65] Lin Q.; Tan X.; Almatrafi E.; Yang Y.; Wang W.; Luo H.; Qin F.; Zhou C.; Zeng G.; Zhang C. Effects of Biochar-Based Materials on the Bioavailability of Soil Organic Pollutants and Their Biological Impacts. Sci. Total Environ. 2022, 826, 153956 10.1016/j.scitotenv.2022.153956. 35189211

[ref66] Yang X.; Hou R.; Fu Q.; Li T.; Li M.; Cui S.; Li Q.; Liu M. A Critical Review of Biochar as an Environmental Functional Material in Soil Ecosystems for Migration and Transformation Mechanisms and Ecological Risk Assessment. J. Environ. Manage. 2024, 360, 121196 10.1016/j.jenvman.2024.121196. 38763117

[ref67] Wang J.; Odinga E. S.; Zhang W.; Zhou X.; Yang B.; Waigi M. G.; Gao Y. Polyaromatic Hydrocarbons in Biochars and Human Health Risks of Food Crops Grown in Biochar-Amended Soils: A Synthesis Study. Environ. Int. 2019, 130, 104899 10.1016/j.envint.2019.06.009. 31203030

[ref68] García-Delgado C.; Delgado-Moreno L.; Toro M.; Puñal M.; Martín-Trueba M.; Eymar E.; Ruíz A. I. The Role of Biochar and Green Compost Amendments in the Adsorption, Leaching, and Degradation of Sulfamethoxazole in Basic Soil. Chemosphere 2023, 344, 140364 10.1016/j.chemosphere.2023.140364. 37797895

[ref69] Hurtado C.; Cañameras N.; Domínguez C.; Price G. W.; Comas J.; Bayona J. M. Effect of Soil Biochar Concentration on the Mitigation of Emerging Organic Contaminant Uptake in Lettuce. J. Hazard. Mater. 2017, 323, 386–393. 10.1016/j.jhazmat.2016.04.046. 27143287

[ref70] Patel A. K.; Katiyar R.; Chen C.-W.; Singhania R. R.; Awasthi M. K.; Bhatia S.; Bhaskar T.; Dong C.-D. Antibiotic Bioremediation by New Generation Biochar: Recent Updates. Bioresour. Technol. 2022, 358, 127384 10.1016/j.biortech.2022.127384. 35644454

[ref71] Sun Y.; Lyu H.; Cheng Z.; Wang Y.; Tang J. Insight into the Mechanisms of Ball-Milled Biochar Addition on Soil Tetracycline Degradation Enhancement: Physicochemical Properties and Microbial Community Structure. Chemosphere 2022, 291, 132691 10.1016/j.chemosphere.2021.132691. 34755608

[ref72] Pan M.; Chu L. M. Fate of Antibiotics in Soil and Their Uptake by Edible Crops. Sci. Total Environ. 2017, 599–600, 500–512. 10.1016/j.scitotenv.2017.04.214. 28482307

[ref73] Cycoń M.; Mrozik A.; Piotrowska-Seget Z. Antibiotics in the Soil Environment—Degradation and Their Impact on Microbial Activity and Diversity. Front. Microbiol. 2019, 10, 338 10.3389/fmicb.2019.00338. 30906284 PMC6418018

[ref74] Carter L. J.; Williams M.; Böttcher C.; Kookana R. S. Uptake of Pharmaceuticals Influences Plant Development and Affects Nutrient and Hormone Homeostases. Environ. Sci. Technol. 2015, 49 (20), 12509–12518. 10.1021/acs.est.5b03468. 26418514

[ref75] Sidhu H.; O’Connor G.; Kruse J. Plant Toxicity and Accumulation of Biosolids-Borne Ciprofloxacin and Azithromycin. Sci. Total Environ. 2019, 648, 1219–1226. 10.1016/j.scitotenv.2018.08.218. 30340267

[ref76] Riemenschneider C.; Seiwert B.; Moeder M.; Schwarz D.; Reemtsma T. Extensive Transformation of the Pharmaceutical Carbamazepine Following Uptake into Intact Tomato Plants. Environ. Sci. Technol. 2017, 51 (11), 6100–6109. 10.1021/acs.est.6b06485. 28506063

[ref77] Tanoue R.; Sato Y.; Motoyama M.; Nakagawa S.; Shinohara R.; Nomiyama K. Plant Uptake of Pharmaceutical Chemicals Detected in Recycled Organic Manure and Reclaimed Wastewater. J. Agric. Food Chem. 2012, 60 (41), 10203–10211. 10.1021/jf303142t. 23003104

[ref78] Gallego S.; Montemurro N.; Béguet J.; Rouard N.; Philippot L.; Pérez S.; Martin-Laurent F. Ecotoxicological Risk Assessment of Wastewater Irrigation on Soil Microorganisms: Fate and Impact of Wastewater-Borne Micropollutants in Lettuce-Soil System. Ecotoxicol. Environ. Saf. 2021, 223, 112595 10.1016/j.ecoenv.2021.112595. 34390984

[ref79] Ponce-Robles L.; Benelhadj L.; García-García A. J.; Pedrero-Salcedo F.; Nortes-Tortosa P. A.; Albacete J.; Alarcón J. J. Risk Assessment for Uptake and Accumulation of Pharmaceuticals by Baby Leaf Lettuce Irrigated with Reclaimed Water under Commercial Agricultural Activities. J. Environ. Manage. 2022, 324, 116321 10.1016/j.jenvman.2022.116321. 36179471

[ref80] Christou A.; Karaolia P.; Hapeshi E.; Michael C.; Fatta-Kassinos D. Long-Term Wastewater Irrigation of Vegetables in Real Agricultural Systems: Concentration of Pharmaceuticals in Soil, Uptake and Bioaccumulation in Tomato Fruits and Human Health Risk Assessment. Water Res. 2017, 109, 24–34. 10.1016/j.watres.2016.11.033. 27865170

[ref81] Zheng W.; Guo M. Soil–Plant Transfer of Pharmaceuticals and Personal Care Products. Curr. Pollut. Rep. 2021, 7 (4), 510–523. 10.1007/s40726-021-00207-2.

[ref82] Margenat A.; You R.; Cañameras N.; Carazo N.; Díez S.; Bayona J. M.; Matamoros V. Occurrence and Human Health Risk Assessment of Antibiotics and Trace Elements in *Lactuca Sativa* Amended with Different Organic Fertilizers. Environ. Res. 2020, 190, 109946 10.1016/j.envres.2020.109946. 32750553

[ref83] Maeusli M.; Lee B.; Miller S.; Reyna Z.; Lu P.; Yan J.; Ulhaq A.; Skandalis N.; Spellberg B.; Luna B. Horizontal Gene Transfer of Antibiotic Resistance from Acinetobacter Baylyi to *Escherichia Coli* on Lettuce and Subsequent Antibiotic Resistance Transmission to the Gut Microbiome. mSphere 2020, 5 (3), e00329-20 10.1128/mSphere.00329-20. 32461272 PMC7253597

[ref84] Bourdat-Deschamps M.; Leang S.; Bernet N.; Daudin J.-J.; Nélieu S. Multi-Residue Analysis of Pharmaceuticals in Aqueous Environmental Samples by Online Solid-Phase Extraction–Ultra-High-Performance Liquid Chromatography-Tandem Mass Spectrometry: Optimisation and Matrix Effects Reduction by Quick, Easy, Cheap, Effective, Rugged and Safe Extraction. J. Chromatogr. A 2014, 1349, 11–23. 10.1016/j.chroma.2014.05.006. 24856968

[ref85] Sun J.; Zeng Q.; Tsang D. C. W.; Zhu L. Z.; Li X. D. Antibiotics in the Agricultural Soils from the Yangtze River Delta, China. Chemosphere 2017, 189, 301–308. 10.1016/j.chemosphere.2017.09.040. 28942256

[ref86] Fang L.; Chen C.; Zhang F.; Ali E. F.; Sarkar B.; Rinklebe J.; Shaheen S. M.; Chen X.; Xiao R. Occurrence Profiling and Environmental Risk Assessment of Veterinary Antibiotics in Vegetable Soils at Chongqing Region, China. Environ. Res. 2023, 227, 115799 10.1016/j.envres.2023.115799. 37015300

[ref87] Ren J.; Shi H.; Liu J.; Zheng C.; Lu G.; Hao S.; Jin Y.; He C. Occurrence, Source Apportionment and Ecological Risk Assessment of Thirty Antibiotics in Farmland System. J. Environ. Manage. 2023, 335, 117546 10.1016/j.jenvman.2023.117546. 36848802

