# Supplementary material for: Assessing Lettuce Exposure to a Multi-Pharmaceutical Mixture in Soil: Insights from LC-ESI-TQ Analysis and the Impact of Biochar on Pharmaceutical Bioavailability
Source: ACS Omega. 2024 Sep 4;9(37):39065–81. doi: 10.1021/acsomega.4c05831 (PMC11411693; doi:10.1021/acsomega.4c05831)
Supplement: Supplementary file 1 — ao4c05831_si_001.pdf [file ao4c05831_si_001.pdf]

Supporting Information for

Assessing Lettuce Exposure to a Multi-Pharmaceutical Mixture in Soil: Insights from LC-ESI-TQ Analysis and the Impact of Biochar on Pharmaceutical Bioavailability

Jan Fučík[1,*], Vojtěch Jašek[2], Marie Hamplová[1], Jitka Navrkalová[1], Helena Zlámalová Gargošová[1], Ludmila Mravcová[1]

[1] *Institute of Chemistry and Technology of Environmental Protection, Faculty of Chemistry, Brno University of Technology, Purkyňova 118, 612 00 Brno, Czech Republic*

[2] *Institute of Materials Chemistry, Faculty of Chemistry, Brno University of Technology, Purkyňova 118, 612 00 Brno, Czech Republic*

*corresponding author: *xcfucikj@vutbr.cz*

Table of Contents

Table S1. Physico-chemical properties of pharmaceuticals

Table S2. Physico-chemical properties of the soil

Table S3. Physico-chemical properties of the biochar

Appendix 1. Extraction methods

Appendix 2. LC-MS/MS method

Table S4. MRM transitions of selected pharmaceuticals for LC-MS/MS analysis

Table S5. Comparison of Time-Weighted Average (TWA) Soil Concentrations with Different Routes of Pharmaceutical Contamination

Figure S1. Heatmap of pharmaceutical distribution in lettuce samples over a 35-day period

Apppendix 3. Calculation Procedure for Hazard Index to Estimate Health Risk

Table S6. Estimation of Health Risk due to Exposure to Pharmaceutical Residues in Lettuce

Appendix 4. Calculation Procedure to Estimate Potential Risk towards Antimicrobial Resistance

Table S7. Risk Quotients towards Emergence of Antimicrobial Resistance

This document contains in total: 17 pages, 7 Tables, 1 Figure

Table S1. Physico-chemical properties of pharmaceuticals, values were sourced from [1,2]

Pharmaceutical group	Pharmaceutical name	Chemical formula	Mw [-]	pKa [-]	log P [-]	Solubility in water [mg·L^{-1}]
BETA BLOCKERS	Acebutolol	$C_{18}H_{28}N_2O_4$	336.4	9.5	1.7	259
FLUOROQUINOLONES	Ciprofloxacin	$C_{17}H_{18}FN_3O_3$	331.3	6.1; 8.7	-1.1	<1
	Enrofloxacin	$C_{19}H_{22}FN_3O_3$	359.4	5.55; 7.24	-0.2	53.9
	Moxifloxacin	$C_{21}H_{24}FN_3O_4$	401.4	6.3; 9.1	0.6	1,146
	Norfloxacin	$C_{16}H_{18}FN_3O_3$	319.3	6.1; 8.8	-1	250,000
	Ofloxacin	$C_{18}H_{20}FN_3O_4$	361.4	6.0; 9.3	-0.4	28,300
	Pefloxacin	$C_{17}H_{20}FN_3O_3$	333.4	5.55; 7.01	0.3	11,400
MACROLIDES	Azithromycin	$C_{38}H_{72}N_2O_{12}$	749.0	8.5	4	2.37
	Clarithromycin	$C_{38}H_{69}NO_{13}$	748.0	9.0	3.2	0.33
	Erythromycin	$C_{37}H_{67}NO_{13}$	733.9	8.9	2.7	4.2
	Roxithromycin	$C_{41}H_{76}N_2O_{15}$	837.0	9.3	1.7	0.0189
SULFONAMIDES AND TRIMETHOPRIM	Sulfacetamide	$C_8H_{10}N_2O_3S$	214.2	2.14; 4.3	-1.0	32.1
	Sulfadiazine	$C_{10}H_{10}N_4O_2S$	250.3	6.4	-0.1	77
	Sulfadimethoxine	$C_{12}H_{14}N_4O_4S$	310.3	1.99; 6.91	1.6	343
	Sulfamerazine	$C_{11}H_{12}N_4O_2S$	264.3	2.0; 6.99	0.1	202
	Sulfamethazine	$C_{12}H_{14}N_4O_2S$	278.3	2.7; 7.7	0.3	1,500
	Sulfamethoxazole	$C_{10}H_{11}N_3O_3S$	253.3	1.6; 5.7	0.9	610
	Sulfamethoxypyridazine	$C_{11}H_{12}N_4O_3S$	280.3	2.02; 6.84	0.3	0.325
	Sulfapyridine	$C_{11}H_{11}N_3O_2S$	249.3	8.4	0	33.1
	Sulfathiazole	$C_9H_9N_3O_2S_2$	255.3	2.2; 7.2	0.1	373
	Trimethoprim	$C_{14}H_{18}N_4O_3$	290.3	7.1	0.9	400
TETRACYCLINES	Chlortetracycline	$C_{22}H_{23}ClN_2O_8$	478.9	7.4	-0.62	0.259
	Doxycycline	$C_{22}H_{24}N_2O_8$	444.4	3.1	-0.7	50
	Oxytetracycline	$C_{22}H_{24}N_2O_9$	460.4	3.3; 9.5	-1.6	47
	Tetracycline	$C_{22}H_{24}N_2O_8$	444.4	3.3; 7.7	-2	231

Table S2. Sampling location and physico-chemical properties of the soil

**Sampling location**	
State	Czech Republic
Region	The Vysočina region
Town	Jemnice
Sampling depth [cm]	0-25
**Physico-chemical properties of soil**	
Soil texture	Sandy Loam
Soil type	Fluvisol
Sand [%]	52.05
Silt [%]	32.65
Clay [%]	15.30
pH $_{(CaCl2)}$ [-]	6.47
pH $_{(H2O)}$ [-]	7.37
EC [mS·cm^{-1}]	0.287
Maximal water holding capacity [%]	44
Exchangable Mg^{2+} [mg·kg-1]	57.1
Exchangable Ca^{2+} [mg·kg^{-1}]	388.2
Exchangable K^+ [mg·kg^{-1}]	135.8
Exchangable Na^+ [mg·kg^{-1}]	45.0
Exchangable NH_4^+ [mg·kg^{-1}]	6.8
Exchangable NO_3^- [mg·kg^{-1}]	189.0
Exchangable PO_4^{3-} [mg·kg^{-1}]	194.2
Total phosphorus – water extract [mg·kg^{-1}]	46.7
Total Nitrogen – water extract [%]	0.128
Total Carbon [%]	2.50
Inorganic Carbon [%]	0.27
Organic carbon [%]	2.23
Organic matter [%]	3.83
Ca - Aqua regia [mg·kg^{-1}]	388.2
K - Aqua regia [mg·kg^{-1}]	1,594
Mg - Aqua regia [mg·kg^{-1}]	7,158
Na - Aqua regia [mg·kg^{-1}]	87.1
NH_4^+ - Aqua regia [mg·kg^{-1}]	89.6
Total phosphorus - Aqua regia [mg·kg^{-1}]	2,822
PO_4^{3-} - Aqua regia [mg·kg^{-1}]	1,973

Table S3. Physico-chemical properties of the biochar, data were obtained from study[3]

**Physico-chemical properties of biochar**	
Dry matter content [%]	65-75
BET [$m^2 \cdot g^{-1}$]	289
$Ash_{550°C}$ [%]	11.7
Total Nitrogen [$g \cdot kg^{-1}$]	13.2
Total Carbon [$g \cdot kg^{-1}$]	866
Organic Carbon [$g \cdot kg^{-1}$]	74
P [$g \cdot kg^{-1}$]	6.2
K [$g \cdot kg^{-1}$]	24.4
Ca [$g \cdot kg^{-1}$]	8.1
Mg [$g \cdot kg^{-1}$]	6.7

Appendix 1. Extraction Methods

QuEChERS Extraction of Lettuce Samples

The lettuce leaves and roots were extracted separately using already validated and published method in previous study.[4] Briefly, 0.1 g of lyophilized and homogenized lettuce leaves were accurately weighed and placed into a 50 mL PE centrifugation tube. In the **extraction step**, ceramic homogenizers were initially introduced, followed by pipetting of 5 mL of the extraction medium (MeOH:McIlvaine buffer, pH 2.6:ACN in a ratio of 8:20:72). Following this, the sample was vortexed for 1 min. Subsequently, separation salts (2 g anhydrous Na_2SO_4 and 0.5 g NaCl) were added, and the mixture was further vortexed for 1.5 min before centrifugation at 3,500 rpm for 10 min at 20 °C. In the **purification step**, following centrifugation, 2 mL of the organic phase was carefully pipetted into a 15 mL PE centrifugation tube preloaded with dSPE sorbents (12.5 mg DSC-18, 12.5 mg PSA, and 225 mg of anhydrous Na_2SO_4). Subsequently, the sample was vortexed for 1 min, followed by another centrifugation at 3,500 rpm for 10 min at 20 °C. Finally, the sample was filtered through 0.22 µm nylon syringe filters (diameter 13 mm) into a 2-mL glass vial, ready for LC-MS/MS analysis.

Extraction of pharmaceuticals from soil

The soil samples were extracted using already validated and published method in previous study.[4] Firstly, 1.0 g of soil was precisely weighed and placed into a 50 mL polyethylene (PE) centrifugation tube. **The extraction procedure (Steps 1-2)** involved pipetting 5 mL of methanol (MeOH) and 5 mL of phosphate buffer (pH 3) into the sample, followed by vortexing for 30 s. Subsequently, PhACs were extracted using an ultrasound bath for 10 min at 12°C. After sonication, the mixture was centrifuged at 4,800 rpm for 8 min at 20 °C. The resulting supernatant was transferred into a 30-mL dark glass vial. The extraction process was then repeated using the same extraction medium and extraction conditions. The extracts obtained from extraction rounds (1-2) were combined and placed in the same vial. In the subsequent **extraction steps (3-4)**, 0.6 g of EDTA was added to the soil along with 7.5 mL of acetonitrile (ACN), 7.5 mL of McIlvaine buffer (pH 8), 4.8 mL of $Mg(NO_3)_2{\cdot}6H_2O$ aqueous solution (concentration 0.5 $g{\cdot}mL^{-1}$), and 0.2 mL of 2.5% NH_3 aqueous solution in the centrifugation tube. The mixture was vortexed for 30 s, followed by PhAC extraction using an ultrasound bath for 10 min at 35°C. After sonication, the solution was centrifuged at 4,800 rpm for 8 min at 20 °C. Subsequently, the supernatant was transferred to a dark 30 mL glass vial. The extraction process was repeated with half the volume of the extraction medium (EM) without the addition of EDTA (3.75 mL of McIlvaine buffer, 3.75 mL of ACN, 2.4 mL of $Mg(NO_3)_2$ solution, and 0.1 mL of 2.5% NH_3) under the same extraction conditions. The extracts obtained from extraction rounds (3-4) were combined and placed in the same vial. During the **pre-concentration step**, both vials were

subjected to evaporation under a nitrogen stream in a thermostatic metal block heated to 40°C until each vial's weight loss reached 6 g. Subsequently, the contents of both vials were combined and transferred into a 600-mL beaker, and the soil extract was diluted by adding 480-mL Milli-Q water to decrease the percentage of the organic phase (no additional pH adjustment was made). **In the solid phase extraction step**, the pre-concentrated soil extracts were purified using Chromservis HLB cartridges (200 mg; 6 mL; Particle diameter 25-35 µm; Chromservis Czech Republic) using a Baker vacuum system (J.T. Baker, Deventer, The Netherlands). To outline the procedure briefly, the SPE column was conditioned with 6 mL of MeOH, followed by 6 mL of Milli-Q water, with a flow rate of approximately 1 $mL \cdot min^{-1}$. Subsequently, the diluted soil extract was loaded onto the column at a flow rate of 5 $mL \cdot min^{-1}$.The washing step was performed with 15 mL of Milli-Q water at a flow rate of 1 $mL \cdot min^{-1}$, followed by 2 min of vacuum drying of the sorbents. Finally, the elution of PhACs was achieved by passing 9 mL of 0.1% formic acid (FA) in MeOH, and the eluate was collected into 20 mL glass vials. This was followed by evaporation under the nitrogen stream in the thermostatic metal block heated to 40°C to dryness. Subsequently, 5 µL of an internal standards mixture (concentration of mixture 10 $\mu g \cdot mL^{-1}$) was introduced, followed by the addition of 995 µL of 0.1% FA in H_2O:ACN (95:5, v/v). The sample was filtered through 0.22 µm nylon syringe filters (diameter 13 mm) into a 2-mL glass vial. The prepared sample was then subjected to LC-MS/MS analysis.

Appendix 2. LC-MS/MS method

Both lettuce and soil sample extracts were analyzed using a previously validated and published method for over 40 pharmaceuticals in previous study.[4] However, in this study, only 20 pharmaceuticals were analyzed, which allowed for lower limits of detection (LoDs) and quantification (LoQs) to be achieved, while maintaining similar recovery rates, as the extraction method remained the same.

Instrumental analysis for the quantification of PhACs in lettuce and soil extracts was performed using ultra-performance liquid chromatography (UHPLC Agilent 1290 Infinity LC) coupled with a triple quadrupole mass spectrometer (Bruker EVOQ LC-TQ) with electrospray ionization (ESI). The gas sources of nitrogen and air were provided by an external gas generator (Peak Scientific – Genius 3045).

Chromatographic separation was accomplished using a Luna Omega Polar C18 Phenomenex column (100 x 2.1 mm, 1.6 µm). The column temperature was optimized at 35°C, and the flow rate was set to 0.5 $mL \cdot min^{-1}$. The mobile phases consisted of A) 0.1% FA in H_2O and B) ACN, following a gradient program for the A eluent (%): t(0 min) = 90, t(0.5 min) = 90, t(13.0 min) = 35, t(14.0 min) = 10, and t(15.5 min) = 90. The LC method was

set to a stop time of 16 min, with a 2-min re-equilibration time. The injection volume for all analyses was 7 µL. To prevent carry-over, an external needle wash was performed using a wash solvent composed of FA:H_2O:ACN at a ratio of 1:9:90 for 30 s.

The MS conditions were set as follows for electrospray ionization in positive mode: spray voltage: 4,500 V; cone temperature: 350°C; cone gas flow: 15 arbitrary units (a.u.); heated probe temperature: 500°C; probe gas flow: 25 a.u.; nebulizer gas flow: 45 a.u.; and exhaust gas: ON. For both quantitative and qualitative analysis of PhACs, the multiple reaction monitoring (MRM) mode was employed, using the specific MRM transitions outlined in Table S4. Argon served as the collision gas at a pressure of 1.5 mTorr.

Table S4. MRM transitions of selected pharmaceuticals for LC-MS/MS analysis

Analyte Name	RT [min]	Quantitative transition			Quantitative transition			Internal standard
		Precursor ion [m/z]	Productn ion [m/z]	CE [eV]	Precursor ion [m/z]	Product ion [m/z]	CE [eV]	
Acebutolol	3.65	337.2	116.0	20.0	337.2	319.0	10.0	Atenolol-d7
Ciprofloxacin	3.35	332.4	314.2	10.0	332.4	288.2	10.0	Ciprofloxacin-d8
Ciprofloxacin-d8	3.35	340.4	322.1	15.0	340.4	296.1	15.0	-
Enrofloxacin	3.78	360.4	316.2	10.0	360.4	342.2	20.0	Enrofloxacin-d5
Enrofloxacin-d5	3.78	365.4	321.0	10.0	365.4	346.8	20.0	-
Moxifloxacin	4.83	402.0	358.2	20.0	402.0	384.2	20.0	Enrofloxacin-d5
Norfloxacin	3.22	320.1	276.1	10.0	320.1	231.1	40.0	Ciprofloxacin-d8
Ofloxacin	3.25	362.2	318.1	10.0	362.2	261.1	20.0	Enrofloxacin-d5
Pefloxacin	3.34	334.0	317.0	10.0	334.0	289.0	10.0	Enrofloxacin-d5
Azithromycin	4.84	375.2	591.3	10.0	375.2	158.0	20.0	Spiramycin
Clarithromycin	7.54	748.5	158.1	20.0	748.5	590.4	10.0	Spiramycin
Erythromycin	6.57	734.5	158.2	30.0	734.5	576.3	10.0	Spiramycin
Roxithromycin	8.03	837.5	679.5	20.0	837.5	157.9	30.0	Spiramycin
Spiramycin	4.78	422.8	699.5	5.0	422.8	540.5	5.0	-
Sulfacetamide	1.62	215.2	156.0	5.0	215.2	108.2	10.0	Sulfamethoxazole-d4
Sulfadiazine	1.96	251.3	156.0	10.0	251.3	92.2	20.0	Sulfamethoxazole-d4
Sulfadimethoxine	5.48	255.0	156.0	10.0	255.0	92.1	15.0	Sulfamethoxazole-d4
Sulfamerazine	2.51	265.3	156.0	10.0	265.3	172.0	10.0	Sulfamethoxazole-d4
Sulfamethazine	3.02	279.3	186.0	10.0	279.3	92.0	30.0	Sulfamethoxazole-d4
Sulfamethoxazole	4.14	254.3	156.0	10.0	254.3	108.1	20.0	Sulfamethoxazole-d4
Sulfamethoxazole-d4	4.12	258.3	159.8	10.0	258.3	96.2	20.0	-
Sulfamethoxypyridazine	3.20	281.1	156.0	10.0	281.1	92.2	20.0	Sulfamethoxazole-d4
Sulfapyridine	1.97	250.3	156.0	10.0	250.3	92.2	20.0	Sulfamethoxazole-d4
Sulfathiazole	2.27	256.0	156.0	10.0	256.0	92.2	20.0	Sulfathiazole-d4
Sulfathiazole-d4	2.23	260.0	96.0	20.0	260.0	160.0	10.0	-
Trimethoprim	2.68	291.2	230.1	20.0	291.2	261.0	20.0	Trimethoprim-d9
Trimethoprim-d9	2.68	300.1	123.0	20.0	300.1	233.8	20.0	-
Chlortetracycline	4.39	479.0	462.3	10.0	479.0	443.7	20.0	none
Doxycycline	5.00	445.4	428.1	10.0	445.4	320.9	30.0	none
Oxytetracycline	2.85	461.0	426.2	20.0	461.0	443.2	5.0	none
Tetracycline	5.00	445.0	427.2	10.0	445.0	428.0	10.0	none

Table S5. Comparison of Time-Weighted Average (TWA) Soil Concentrations with Different Routes of Pharmaceutical Contamination

Pharmaceutical Group	Pharmaceutical Name	TWA soil concentration in [$ng \cdot g^{-1}$] for initialy spiked soil (C_0 = 70 $ng \cdot g^{-1}$)	TWA soil concentration in [$ng \cdot g^{-1}$] for irrigated soil with contaminated water (C_0 = 50 $\mu g \cdot L^{-1}$)
beta-blockers	acetobutolol	44.10	25.69
fluoroquinolones	ciprofloxacin	23.67	16.71
	enrofloxacin	61.71	32.18
	moxifloxacin	58.67	31.11
	norfloxacin	59.85	31.53
	ofloxacin	55.84	30.10
	pefloxacin	20.71	15.17
macrolides	azihromycin	69.26	34.75
	clarithromycin	40.24	24.15
	erythromycin	29.81	19.66
	roxithromycin	23.87	16.81
sulfonamides	sulfacetamide	12.58	10.36
	sulfadiazine	23.60	16.67
	sulfadimethoxine	37.67	23.09
	sulfamerazine	35.71	22.27
	sulfamethazine	36.87	22.76
	sulfamethoxazole	42.59	25.10
	sulfamethoxypyridazine	29.53	19.53
	sulfapyridine	18.73	14.09
	sulfathiazole	18.34	13.87
	trimethoprim	52.26	28.8
tetracyclines	chlortetracycline	43.59	25.49
	doxycycline	47.36	26.96
	oxytetracycline	48.52	27.40
	tetracycline	46.72	26.71

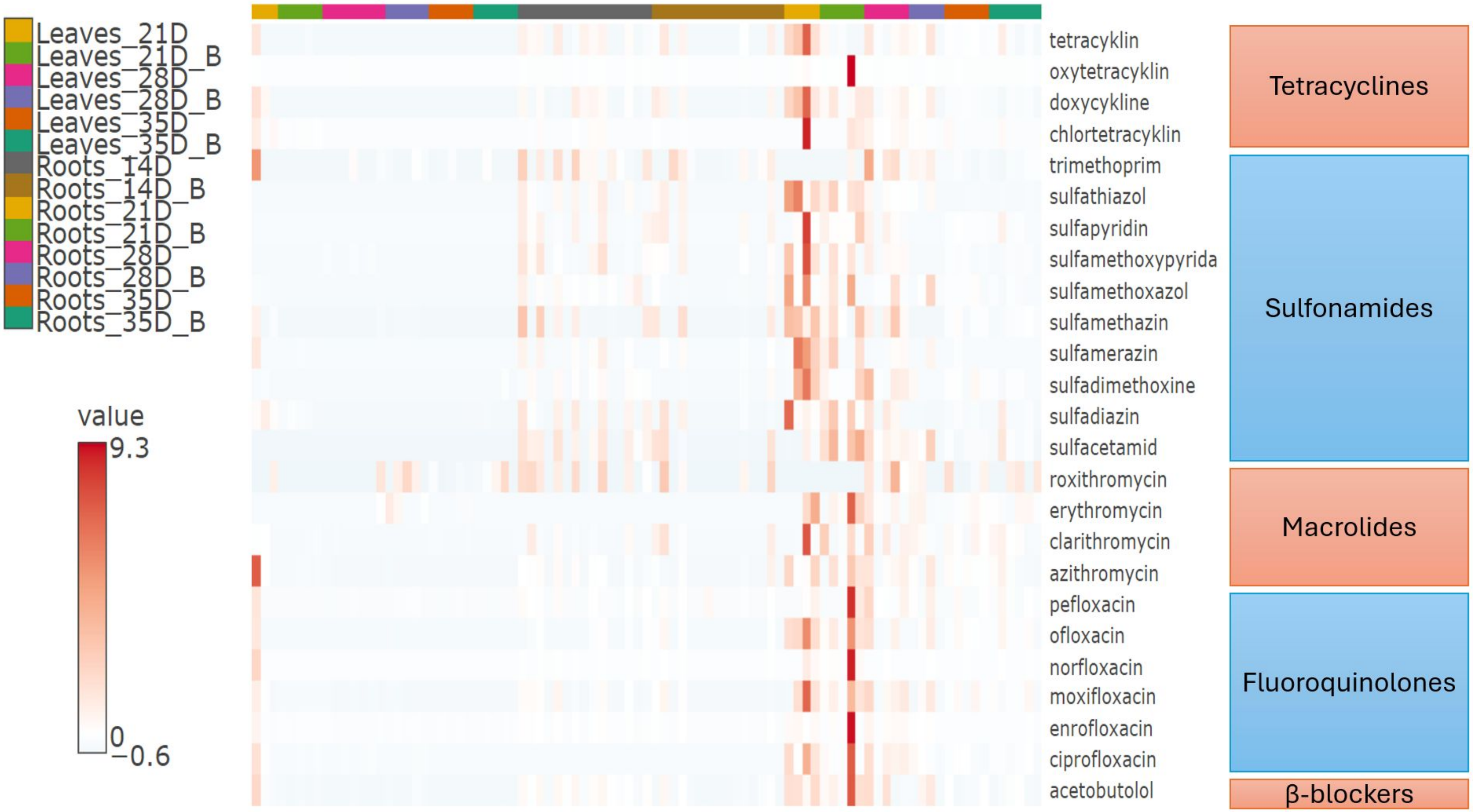

Figure S1. Heatmap of pharmaceutical distribution in lettuce samples over a 35-day period

Appendix 3. Calculation Procedure for Hazard Index to Estimate Health Risk

The estimated daily intake (EDI) for each pharmaceutical was calculated using time-weighted average soil concentrations, as detailed in the Main Manuscript, subsection 3.1. We derived the EDI for different soil treatments (soil spiking vs soil irrigation) at two concentration levels for each treatment type (soil spiking at 100 and 1,000 $ng{\cdot}g^{-1}$, and soil irrigation at 5 and 50 $\mu g{\cdot}L^{-1}$). To estimate pharmaceutical concentrations in lettuce leaves, we used Eq. 1, taking into account soil treatments and concentrations, and applied the calculated bioaccumulation factors (BCFs) and translocation factors (TFs) from the main manuscript, as detailed in the Main Manuscript, subsection 3.1. The concentration of pharmaceuticals in lettuce leaves was then used in Eq. 2 to estimate the EDI for adult individuals, assuming an average body weight of 70 kg.[5,6] The average water content in lettuce leaves is approximately 95%, which aligns with previous studies.[6,7] The average daily intake rate of lettuce is 8.57 g per person per day, as reported in study.[8]

$$C_{\text{lettuce leaves}} = \text{BCF} \cdot \text{TF} \cdot C_{soil\ avg.} \qquad (1)$$

where $C_{lettuce\ leaves}$ [$ng{\cdot}g^{-1}$ dw] represents the concentration of pharmaceutical in lettuce leaves. BCF [-] stands for bioconcentration factor, indicating the ratio of the pharmaceutical concentration in lettuce roots to the soil. TF [-] represents the translocation factor, measuring the extent to which pharmaceuticals are transferred from lettuce roots to leaves. $C_{\text{soil avg.}}$ [$ng{\cdot}g^{-1}$ dw] denotes the time-weighted average concentration of the pharmaceutical in the soil.

$$\text{EDI} = \frac{C_{\text{lettuce leaves}} \cdot \beta_{fw/dw} \cdot IR_{lettuce}}{m_{body\ weight}} \qquad (2)$$

where EDI [$ng{\cdot}kg^{-1}{\cdot}day^{-1}$] stands for estimated daily intake, $C_{\text{lettuce leaves}}$ [$ng{\cdot}g^{-1}$ dw] represents the concentration of pharmaceutical in lettuce leaves, $\beta_{fw/dw}$ [-] denotes the conversion factor from dry weight to fresh weight, IR_{lettuce} [$g{\cdot}day^{-1}$] is the average daily intake of lettuce and $m_{\text{body weight}}$ [kg] indicates the average body weight of an adult.

Consequently, we searched scientific literature for acceptable daily intake (ADI) values [9–14] to establish benchmarks for calculating risk quotients (RQs) (Table S6). Using these values, we calculated the RQ for each pharmaceutical separately with Eq. 3. Next, we computed the Hazard Index (HI) as the sum of all RQs for each soil treatment using Eq. 4 (Table S6), as described in previous studies.[6,15] A human health risk is considered negligible when the RQ or HI is less than 0.01, considerable when either RQ or HI exceeds 0.01, and distinct when either value is greater than 0.05.[15]

$$\mathrm{RQ} = \frac{EDI}{ADI} \tag{3}$$

where RQ [-] is risk quotient, EDI [ng·kg^{-1}·day^{-1}] is estimated daily intake and ADI [ng·kg^{-1}·day^{-1}] stands for acceptable daily intake.

$$HI = \sum RQ \tag{4}$$

where HI [-] stands for hazard index and RQ [-] denotes risk quotient.

Table S6. Estimation of Health Risk due to Exposure to Pharmaceutical Residues in Lettuce - Acceptable daily intake (ADI) for selected pharmaceuticals, their estimated daily intake (EDI) at different soil treatments (soil spike or soil irrigation), with calculated risk quotients (RQ) for each compound and total sum of RQs as hazard risk (HI) at different levels of soil contamination [9–14,16], N.D. – not determined.

Pharmaceutical Group	Pharmaceutical Name	ADI [$\mu g \cdot kg^{-1} \cdot day^{-1}$]	EDI 100 $ng \cdot g^{-1}$ soil spike [$ng \cdot kg^{-1} \cdot day^{-1}$]	RQ 100 $ng \cdot g^{-1}$ soil spike [-]	EDI 1,000 $ng \cdot g^{-1}$ soil spike [$ng \cdot kg^{-1} \cdot day^{-1}$]	RQ 1,000 $ng \cdot g^{-1}$ soil spike [-]	EDI 5 $\mu g \cdot L^{-1}$ soil irrigation [$ng \cdot kg^{-1} \cdot day^{-1}$]	RQ 5 $\mu g \cdot L^{-1}$ soil irrigation [-]	EDI 50 $\mu g \cdot L^{-1}$ soil irrigation [$ng \cdot kg^{-1} \cdot day^{-1}$]	RQ 50 $\mu g \cdot L^{-1}$ soil irrigation [-]
beta-blockers	acetobutolol	N.D.	0.016	N.D.	0.1624	N.D.	0.0055	N.D.	0.0547	N.D.
fluoroquinolones	ciprofloxacin	1.6	0.015	9.68E-06	0.1549	9.68E-05	0.0025	1.58E-06	0.0253	1.58E-05
	enrofloxacin	2.0	0.013	6.56E-06	0.1313	6.56E-05	N.D.	N.D.	N.D.	N.D.
	moxifloxacin	N.D.	0.028	N.D.	0.2849	N.D.	0.0109	N.D.	0.1089	N.D.
	norfloxacin	11.4	N.D.	N.D.	N.D.	N.D.	N.D.	N.D.	N.D.	N.D.
	ofloxacin	5.7	0.012	2.04E-06	0.1160	2.04E-05	0.0051	8.98E-07	0.0512	8.98E-06
	pefloxacin	N.D.	0.011	N.D.	0.1065	N.D.	0.0107	N.D.	0.1065	N.D.
macrolides	azithromycin	1.7	0.006	3.74E-06	0.0635	3.74E-05	0.0037	2.17E-06	0.0369	2.17E-05
	clarithromycin	0.2	0.003	1.74E-05	0.0348	1.74E-04	0.0027	1.36E-05	0.0272	1.36E-04
	erythromycin	0.7	0.129	1.84E-04	1.2850	1.84E-03	0.0025	3.58E-06	0.0250	3.58E-05
	roxithromycin	0.4	0.026	6.60E-05	0.2641	6.60E-04	N.D.	N.D.	N.D.	N.D.
sulfonamides	sulfacetamide	N.D.	N.D.	N.D.	N.D.	N.D.	N.D.	N.D.	N.D.	N.D.
	sulfadiazine	20.0	N.D.	N.D.	N.D.	N.D.	N.D.	N.D.	N.D.	N.D.
	sulfadimethoxine	10.0	N.D.	N.D.	N.D.	N.D.	N.D.	N.D.	N.D.	N.D.
	sulfamerazine	10.0	N.D.	N.D.	N.D.	N.D.	N.D.	N.D.	N.D.	N.D.
	sulfamethazine	10.0	N.D.	N.D.	N.D.	N.D.	N.D.	N.D.	N.D.	N.D.
	sulfamethoxazole	5.7	N.D.	N.D.	N.D.	N.D.	0.0034	5.91E-07	0.0338	5.91E-06
	sulfamethoxypyridazine	N.D.	0.018	N.D.	0.1772	N.D.	N.D.	N.D.	N.D.	N.D.
	sulfapyridine	3.6	0.005	1.50E-06	0.0537	1.50E-05	N.D.	N.D.	N.D.	N.D.
	sulfathiazole	50.0	N.D.	N.D.	N.D.	N.D.	N.D.	N.D.	N.D.	N.D.
	trimethoprim	4.0	0.022	5.44E-06	0.2176	5.44E-05	0.0027	6.82E-07	0.0273	6.82E-06
tetracyclines	chlortetracycline	50.0	0.011	2.18E-07	0.1089	2.18E-06	0.0039	7.73E-08	0.0387	7.73E-07
	doxycycline	3.0	N.D.	N.D.	N.D.	N.D.	0.0123	4.12E-06	0.1235	4.12E-05
	oxytetracycline	30.0	0.014	4.71E-07	0.1412	4.71E-06	N.D.	N.D.	N.D.	N.D.
	tetracycline	5.7	N.D.	N.D.	N.D.	N.D.	N.D.	N.D.	N.D.	N.D.
**Estimation of Health Risk due to Exposure to Pharmaceutical Residues in Lettuce:**			HI 100 $ng \cdot g^{-1}$ soil spike [-]	0.0003	HI 1,000 $ng \cdot g^{-1}$ soil spike [-]	0.0030	HI 5 $\mu g \cdot L^{-1}$ soil irrigation [-]	0.00003	HI 50 $\mu g \cdot L^{-1}$ soil irrigation [-]	0.00027

Appendix 4. Calculation Procedure to Estimate Potential Risk towards Antimicrobial Resistance

The range of risk quotients (RQs) towards the emergence of antimicrobial resistance in the soil environment were assessed at day 0 and after 28 days for various soil treatments and concentrations. Specifically, soil was spiked at concentrations of 100 and 1,000 $ng \cdot g^{-1}$, while soil irrigation was conducted at concentrations of 5 and 50 $\mu g \cdot L^{-1}$. RQ values were determined as the ratio of measured environmental concentrations (MEC) to predicted no-effect concentrations (PNEC).

Since PNEC-MIC (Predicted No-Effect Concentration - Minimum Inhibitory Concentration) soil values were not available in the literature, PNEC-MIC water values were sourced from the document [17], providing insights into the potential risk of antimicrobial resistance in aquatic environments (Table S7). Subsequently, PNEC-MIC soil values were calculated using Eq. 5. This involved obtaining distribution coefficients (*Kd*) from relevant studies [18–26], acknowledging their dependence on soil physicochemical properties. The determination of ranges for both Kd and PNEC-MIC soil values facilitated the estimation of potential risks associated with antimicrobial resistance in terrestrial environments (Table S7). Furthermore, to account for the co-occurrence of multiple veterinary antimicrobials in soils, we calculated the cumulative risk quotient (∑RQ) to assess the comprehensive ecological risks associated with antimicrobial resistance.[27]

The criteria for interpreting RQs were as commonly used: low risk when RQ < 0.1, medium risk when 0.1 < RQ < 1, and high risk for RQ > 1.[28,29]

$$PNEC_{soil} = PNEC_{water} \cdot Kd \quad (5)$$

where $PNEC_{soil}$ [$ng \cdot g^{-1}$] is the predicted no-effect concentration in soil, $PNEC_{water}$ [$\mu g \cdot L^{-1}$] is the predicted no-effect concentration, and Kd [$L \cdot kg^{-1}$] is the distribution coefficient for a given compound in the soil environment.

Table S7. Risk Quotients towards Emergence of Antimicrobial Resistance (PNEC-MIC water and Kd values were obtained from [17–26]

Pharmaceutical Group	Pharmaceutical Name	PNEC-MIC water [μg·L^{-1}]	Kd [L·kg^{-1}]	PNEC-MIC soil [ng·g^{-1}] [calc. Eq 5]	Range of Risk Quotients towards Emergence of Antimicrobial Resistance							
					Soil Spike 100 ng·g^{-1}		Soil Spike 1,000 ng·g^{-1}		Soil Irrigation 5 μg·L^{-1}		Soil Irrigation 50 μg·L^{-1}	
					0 day	28 days	0 day	28 days	0 day	28 days	0 day	28 days
fluoroquinolones	ciprofloxacin	0.06	427-430	25.6-25.8	3.88-3.90	0.242-0.244	38.8-39.0	2.42-2.44	0.0	0.0918-0.0924	0.0	0.918-0.924
	enrofloxacin	0.06	260-6,310	15.6-379	0.26-6.41	0.2-5.0	2.6-64	2.0-49.6	0.0	0.016-0.396	0.0	0.16-3.96
	moxifloxacin	0.13	3,018	392	0.25	0.177	2.55	1.77	0.0	0.015	0.0	0.150
	norfloxacin	0.5	591-5,791	296-2,896	0.03-0.34	0.025-0.245	0.35-3.38	0.25-2.45	0.0	0.002-0.020	0.0	0.02-0.20
	ofloxacin	0.5	309-4,325	155-2,163	0.05-0.65	0.03-0.40	0.46-6.47	0.29-4.04	0.0	0.003-0.036	0.0	0.03-0.36
	pefloxacin	8	N.D.	N.D.	N.D.	N.D.	N.D.	N.D.	0.0	N.D.	0.0	N.D.
macrolides	azithromycin	0.25	37-347	9.3-86.8	1.2-10.8	1.1-10.6	11.5-108	11.28-106	0.0	0.08-0.75	0.0	0.8-7.5
	clarithromycin	0.25	2.5-10.5	0.6-2.6	38-160	11-47	381-1,600	111-467	0.0	1.5-6.4	0.0	15-64
	erythromycin	1	67.6-337	67.6-337	0.3-1.5	0.038-0.192	3.0-15.0	0.38-1.92	0.0	0.009-0.044	0.0	0.09-0.44
	roxithromycin	1	667-2,392	667-2,392	0.04-0.15	0.003-0.010	0.42-1.50	0.03-0.10	0.0	0.001-0.004	0.0	0.01-0.04
sulfonamides	sulfacetamide	N.D.	N.D.	N.D.	N.D.	N.D.	N.D.	N.D.	0.0	N.D.	0.0	N.D.
	sulfadiazine	N.D.	2.5	N.D.	N.D.	N.D.	N.D.	N.D.	0.0	N.D.	0.0	N.D.
	sulfadimethoxine	N.D.	2.3-10	N.D.	N.D.	N.D.	N.D.	N.D.	0.0	N.D.	0.0	N.D.
	sulfamerazine	N.D.	N.D:	N.D.	N.D.	N.D.	N.D.	N.D.	0.0	N.D.	0.0	N.D.
	sulfamethazine	N.D.	0.6-3.1	N.D.	N.D.	N.D.	N.D.	N.D.	0.0	N.D.	0.0	N.D.
	sulfamethoxazole	16	8.1-59.4	130-950	0.11-0.77	0.035-0.259	1.05-7.72	0.35-2.59	0.0	0.005-0.033	0.0	0.05-0.33
	sulfamethoxypyridazine	N.D.	N.D.	N.D.	N.D.	N.D.	N.D.	N.D.	0.0	N.D.	0.0	N.D.
	sulfapyridine	N.D.	1.6-7.4	N.D.	N.D.	N.D.	N.D.	N.D.	0.0	N.D.	0.0	N.D.
	sulfathiazole	N.D.	3.0-4.9	N.D.	N.D.	N.D.	N.D.	N.D.	0.0	N.D.	0.0	N.D.
	trimethoprim	0.5	10.0-104.6	5.0-52.3	1.9-20.0	1.0-10.8	19-200	10.3-108	0.0	0.10-1.05	0.0	1.0-10.5
tetracyclines	chlortetracycline	N.D.	282-2,608	N.D.	N.D.	N.D.	N.D.	N.D.	0.0		0.0	N.D.
	doxycycline	2	431-11,908	862-23,816	0.004-0.12	0.002-0.053	0.04-1.16	0.02-0.53	0.0	0.0002-0.0056	0.0	0.002-0.056
	oxytetracycline	0.5	417-1,026	209-513	0.19-0.48	0.089-0.219	1.95-4.80	0.89-2.19	0.0	0.010-0.023	0.0	0.095-0.233
	tetracycline	1	198-1,620	198-1,620	0.06-0.51	0.026-0.211	0.62-5.05	0.26-2.11	0.0	0.003-0.024	0.0	0.03-0.24
**Accumulative Risk Quotients**				**∑RQ**	46-206	14-75	463-2,058	141-750	0.0	1.87-8.93	0.0	19-89

REFERENCES

(1) Kim, S.; Chen, J.; Cheng, T.; Gindulyte, A.; He, J.; He, S.; Li, Q.; Shoemaker, B. A.; Thiessen, P. A.; Yu, B.; Zaslavsky, L.; Zhang, J.; Bolton, E. E. PubChem 2023 Update. *Nucleic Acids Res* **2023**, *51* (D1), D1373–D1380. https://doi.org/10.1093/nar/gkac956.

(2) Knox, C.; Wilson, M.; Klinger, C. M.; Franklin, M.; Oler, E.; Wilson, A.; Pon, A.; Cox, J.; Chin, N. E. (Lucy); Strawbridge, S. A.; Garcia-Patino, M.; Kruger, R.; Sivakumaran, A.; Sanford, S.; Doshi, R.; Khetarpal, N.; Fatokun, O.; Doucet, D.; Zubkowski, A.; Rayat, D. Y.; Jackson, H.; Harford, K.; Anjum, A.; Zakir, M.; Wang, F.; Tian, S.; Lee, B.; Liigand, J.; Peters, H.; Wang, R. Q. (Rachel); Nguyen, T.; So, D.; Sharp, M.; da Silva, R.; Gabriel, C.; Scantlebury, J.; Jasinski, M.; Ackerman, D.; Jewison, T.; Sajed, T.; Gautam, V.; Wishart, D. S. DrugBank 6.0: The DrugBank Knowledgebase for 2024. *Nucleic Acids Res* **2024**, *52* (D1), D1265–D1275. https://doi.org/10.1093/nar/gkad976.

(3) Holatko, J.; Brtnicky, M.; Mustafa, A.; Kintl, A.; Skarpa, P.; Ryant, P.; Baltazar, T.; Malicek, O.; Latal, O.; Hammerschmiedt, T. Effect of Digestate Modified with Amendments on Soil Health and Plant Biomass under Varying Experimental Durations. *Materials* **2023**, *16* (3), 1027. https://doi.org/10.3390/ma16031027.

(4) Mravcová, L.; Amrichová, A.; Navrkalová, J.; Hamplová, M.; Sedlář, M.; Gargošová, H. Z.; Fučík, J. Optimization and Validation of Multiresidual Extraction Methods for Pharmaceuticals in Soil, Lettuce, and Earthworms. *Environmental Science and Pollution Research* **2024**. https://doi.org/10.1007/s11356-024-33492-7.

(5) Kodešová, R.; Klement, A.; Golovko, O.; Fér, M.; Kočárek, M.; Nikodem, A.; Grabic, R. Soil Influences on Uptake and Transfer of Pharmaceuticals from Sewage Sludge Amended Soils to Spinach. *J Environ Manage* **2019**, *250*, 109407. https://doi.org/10.1016/j.jenvman.2019.109407.

(6) Geng, J.; Liu, X.; Wang, J.; Li, S. Accumulation and Risk Assessment of Antibiotics in Edible Plants Grown in Contaminated Farmlands: A Review. *Science of The Total Environment* **2022**, *853*, 158616. https://doi.org/10.1016/j.scitotenv.2022.158616.

(7) Wu, X.; Ernst, F.; Conkle, J. L.; Gan, J. Comparative Uptake and Translocation of Pharmaceutical and Personal Care Products (PPCPs) by Common Vegetables. *Environ Int* **2013**, *60*, 15–22. https://doi.org/10.1016/j.envint.2013.07.015.

(8) Hospido, A.; Milà i Canals, L.; McLaren, S.; Truninger, M.; Edwards-Jones, G.; Clift, R. The Role of Seasonality in Lettuce Consumption: A Case Study of Environmental and Social Aspects. *Int J Life Cycle Assess* **2009**, *14* (5), 381–391. https://doi.org/10.1007/s11367-009-0091-7.

(9) Liu, S.; Zhao, H.; Lehmler, H.-J.; Cai, X.; Chen, J. Antibiotic Pollution in Marine Food Webs in Laizhou Bay, North China: Trophodynamics and Human Exposure Implication. *Environ Sci Technol* **2017**, *51* (4), 2392–2400. https://doi.org/10.1021/acs.est.6b04556.

(10) Ji, K.; Kho, Y.; Park, C.; Paek, D.; Ryu, P.; Paek, D.; Kim, M.; Kim, P.; Choi, K. Influence of Water and Food Consumption on Inadvertent Antibiotics Intake among General Population. *Environ Res* **2010**, *110* (7), 641–649. https://doi.org/10.1016/j.envres.2010.06.008.

(11) Subirats, J.; Domingues, A.; Topp, E. Does Dietary Consumption of Antibiotics by Humans Promote Antibiotic Resistance in the Gut Microbiome? *J Food Prot* **2019**, *82* (10), 1636–1642. https://doi.org/10.4315/0362-028X.JFP-19-158.

(12) Hanna, N.; Sun, P.; Sun, Q.; Li, X.; Yang, X.; Ji, X.; Zou, H.; Ottoson, J.; Nilsson, L. E.; Berglund, B.; Dyar, O. J.; Tamhankar, A. J.; Stålsby Lundborg, C. Presence of Antibiotic Residues in Various Environmental Compartments of Shandong Province in Eastern China: Its Potential for Resistance Development and Ecological and Human Risk. *Environ Int* **2018**, *114*, 131–142. https://doi.org/10.1016/j.envint.2018.02.003.

(13) Prosser, R. S.; Sibley, P. K. Human Health Risk Assessment of Pharmaceuticals and Personal Care Products in Plant Tissue Due to Biosolids and Manure Amendments, and Wastewater Irrigation. *Environ Int* **2015**, *75*, 223–233. https://doi.org/10.1016/j.envint.2014.11.020.

(14) Liu, X.; Liang, C.; Liu, X.; Zhao, F.; Han, C. Occurrence and Human Health Risk Assessment of Pharmaceuticals and Personal Care Products in Real Agricultural Systems with Long-Term Reclaimed Wastewater Irrigation in Beijing, China. *Ecotoxicol Environ Saf* **2020**, *190*, 110022. https://doi.org/10.1016/j.ecoenv.2019.110022.

(15) Keerthanan, S.; Jayasinghe, C.; Biswas, J. K.; Vithanage, M. Pharmaceutical and Personal Care Products (PPCPs) in the Environment: Plant Uptake, Translocation, Bioaccumulation, and Human Health Risks. *Crit Rev Environ Sci Technol* **2021**, *51* (12), 1221–1258. https://doi.org/10.1080/10643389.2020.1753634.

(16) Liu, X.; Lu, S.; Meng, W.; Zheng, B. Residues and Health Risk Assessment of Typical Antibiotics in Aquatic Products from the Dongting Lake, China—"Did You Eat 'Antibiotics' Today?" *Environmental Science and Pollution Research* **2018**, *25* (4), 3913–3921. https://doi.org/10.1007/s11356-017-0745-0.

(17) AMR Industry Alliance. *AMR Alliance Science-Based PNEC Targets for Risk Assessments*; 2023.

(18) Gravesen, C.; Judy, J. D. Effect of Biosolids Characteristics on Retention and Release Behavior of Azithromycin and Ciprofloxacin. *Environ Res* **2020**, *184*, 109333. https://doi.org/10.1016/j.envres.2020.109333.

(19) Harrower, J.; McNaughtan, M.; Hunter, C.; Hough, R.; Zhang, Z.; Helwig, K. Chemical Fate and Partitioning Behavior of Antibiotics in the Aquatic Environment—A Review. *Environ Toxicol Chem* **2021**, *40* (12), 3275–3298. https://doi.org/10.1002/etc.5191.

(20) Meng, F.; Sun, S.; Geng, J.; Ma, L.; Jiang, J.; Li, B.; Yabo, S. D.; Lu, L.; Fu, D.; Shen, J.; Qi, H. Occurrence, Distribution, and Risk Assessment of Quinolone Antibiotics in Municipal Sewage Sludges throughout China. *J Hazard Mater* **2023**, *453*, 131322. https://doi.org/10.1016/j.jhazmat.2023.131322.

(21) Pan, M.; Chu, L. M. Adsorption and Degradation of Five Selected Antibiotics in Agricultural Soil. *Science of The Total Environment* **2016**, *545–546*, 48–56. https://doi.org/10.1016/j.scitotenv.2015.12.040.

(22) Chee-Sanford, J. C.; Mackie, R. I.; Koike, S.; Krapac, I. G.; Lin, Y.; Yannarell, A. C.; Maxwell, S.; Aminov, R. I. Fate and Transport of Antibiotic Residues and Antibiotic Resistance Genes Following Land Application of Manure Waste. *J Environ Qual* **2009**, *38* (3), 1086–1108. https://doi.org/10.2134/jeq2008.0128.

(23) Rodríguez-López, L.; Santás-Miguel, V.; Cela-Dablanca, R.; Núñez-Delgado, A.; Álvarez-Rodríguez, E.; Pérez-Rodríguez, P.; Arias-Estévez, M. Ciprofloxacin and Trimethoprim Adsorption/Desorption in Agricultural Soils. *Int J Environ Res Public Health* **2022**, *19* (14), 8426. https://doi.org/10.3390/ijerph19148426.

(24) Rodríguez-López, L.; Santás-Miguel, V.; Cela-Dablanca, R.; Núñez-Delgado, A.; Álvarez-Rodríguez, E.; Rodríguez-Seijo, A.; Arias-Estévez, M. Clarithromycin as Soil and Environmental Pollutant: Adsorption-Desorption Processes and Influence of PH. *Environ Res* **2023**, *233*, 116520. https://doi.org/10.1016/j.envres.2023.116520.

(25) Tang, J.; Wang, S.; Fan, J.; Long, S.; Wang, L.; Tang, C.; Tam, N. F.; Yang, Y. Predicting Distribution Coefficients for Antibiotics in a River Water–Sediment Using Quantitative Models Based on Their Spatiotemporal Variations. *Science of The Total Environment* **2019**, *655*, 1301–1310. https://doi.org/10.1016/j.scitotenv.2018.11.163.

(26) Thiele-Bruhn, S. Pharmaceutical Antibiotic Compounds in Soils – a Review. *Journal of Plant Nutrition and Soil Science* **2003**, *166* (2), 145–167. https://doi.org/10.1002/jpln.200390023.

(27) Fang, L.; Chen, C.; Zhang, F.; Ali, E. F.; Sarkar, B.; Rinklebe, J.; Shaheen, S. M.; Chen, X.; Xiao, R. Occurrence Profiling and Environmental Risk Assessment of Veterinary Antibiotics in Vegetable Soils at Chongqing Region, China. *Environ Res* **2023**, *227*, 115799. https://doi.org/10.1016/j.envres.2023.115799.

(28) Bourdat-Deschamps, M.; Leang, S.; Bernet, N.; Daudin, J.-J.; Nélieu, S. Multi-Residue Analysis of Pharmaceuticals in Aqueous Environmental Samples by Online Solid-Phase Extraction–Ultra-High-Performance Liquid Chromatography-Tandem Mass Spectrometry: Optimisation and Matrix Effects Reduction by Quick, Easy, Cheap, Effective, Rugged and Safe Extraction. *J Chromatogr A* **2014**, *1349*, 11–23. https://doi.org/10.1016/j.chroma.2014.05.006.

(29) Sun, J.; Zeng, Q.; Tsang, D. C. W.; Zhu, L. Z.; Li, X. D. Antibiotics in the Agricultural Soils from the Yangtze River Delta, China. *Chemosphere* **2017**, *189*, 301–308. https://doi.org/10.1016/j.chemosphere.2017.09.040.
